# Proportion and Characteristics of Helicobacter *Pylori*-Negative Gastric Mucosa-Associated Lymphoid Tissue Lymphoma: A Systematic Review and Meta-Analysis

**DOI:** 10.14309/ctg.0000000000000781

**Published:** 2024-10-25

**Authors:** Xiu-He Lv, Qing Lu, Jia-Huan Liu, Bi-Han Xia, Zi-Jing Wang, Zhu Wang, Jin-Lin Yang

**Affiliations:** 1 Department of Gastroenterology and Hepatology, West China Hospital of Sichuan University, Chengdu, Sichuan, China;; 2 Department of Gastroenterology and Hepatology, Sichuan University-Oxford University Huaxi Gastrointestinal Cancer Centre, West China Hospital of Sichuan University, Chengdu, Sichuan, China.

**Keywords:** *Helicobacter pylori*, mucosa-associated lymphoid tissue lymphoma, risk factor, meta-analysis

## Abstract

**INTRODUCTION:**

While *Helicobacter pylori* (*H*. *pylori*) infection is common in patients with gastric mucosa-associated lymphoid tissue (MALT) lymphoma, there are still individuals who test negative for it. The proportion and characteristics of these patients remain unclear.

**METHODS:**

We conducted a systematic search of the PubMed, Embase, and Cochrane Library databases for relevant articles. Using a random-effects model, we performed a meta-analysis to assess the pooled proportion of gastric MALT lymphoma patients with negative *H. pylori* tests. In addition, we compared characteristics between gastric MALT lymphoma patients with and without *H. pylori* infection to examine clinical features in *H. pylori*-negative cases.

**RESULTS:**

A total of 50 studies involving 6,033 patients were included. The overall proportion of gastric MALT lymphoma patients with negative *H. pylori* tests was 20.5% (95% confidence interval: 17.0%–24.6%). This rate exhibited an increasing trend over the years, particularly in non-Asian countries and in studies published after 2013, as well as in cases with sample sizes exceeding 100 patients, in male individuals, and among those with proximal or multiple lesions, nonsuperficial type morphology, submucosal invasion, and advanced clinical staging. Compared with *H. pylori*-positive patients, those who tested negative were more likely to be male, have proximal lesions, exhibit submucosal invasion, and present with an advanced clinical stage.

**DISCUSSION:**

This study provides comprehensive information on the proportion and characteristics of *H. pylori*-negative gastric MALT lymphoma cases, highlighting the need for future clinical attention to treatment and surveillance in this patient population.

## INTRODUCTION

Gastric mucosa-associated lymphoid tissue (MALT) lymphoma is a rare malignancy that arises from acquired MALT in the stomach, typically due to long-term infection with Helicobacter *pylori* (*H. pylori*) (1). The chronic *H. pylori* infection leads to the accumulation of CD4^+^ lymphocytes and mature B cells in the gastric lamina propria. Antigens from *H. pylori* stimulate T-cell activation, B-cell proliferation, and lymphoid follicle development, which can progress to monoclonal lymphoma if the infection persists (2). Eradication therapy for *H. pylori* has been demonstrated to be effective in treating early-stage gastric MALT lymphomas (3,4). Some studies also suggest that combining radiotherapy or chemotherapy with eradication therapy may enhance the potential for curing gastric MALT lymphoma.

However, not all gastric MALT lymphomas exhibit *H. pylori* positivity. Between 6% and 40% of patients with gastric MALT lymphoma test negative for the bacterium, indicating that their development may be independent of *H. pylori* infection (5). This specific subtype of gastric MALT lymphoma is now believed to be associated with genetic mutations, other bacterial or viral infections, and autoimmune disorders (6). Recent studies also indicate a rising incidence of this particular subtype of gastric MALT lymphoma (7). The optimal treatment approach for *H. pylori*-negative gastric MALT lymphoma remains a topic of debate among medical professionals (8,9). These MALT lymphomas pose multiple challenges due to the limited understanding of their occurrence and clinical features. Therefore, the objective of this study was to rigorously assess the proportion and characteristics of *H. pylori*-negative gastric MALT lymphomas using evidence-based methods.

## METHODS

This study performed a systematic review and meta-analysis using the guidelines outlined in the Preferred Reporting Items for Systematic Reviews and Meta-Analyses Statement (10). The research methodology has been accurately documented in the International Prospective Register of Systematic Reviews database (ID: CRD42024533756).

### Search strategy

A comprehensive literature search was conducted on March 26, 2024, without any limitations on publication dates or languages. We used the PubMed, Embase, and Cochrane library databases to perform a detailed search for relevant studies. The primary search terms included “*Helicobacter pylori*”, “*H. pylori*,” “mucosa-associated lymphoid tissue lymphoma,” “B-cell lymphoma,” and “marginal-zone lymphoma” (see Table S1, Supplementary Digital Content, http://links.lww.com/CTG/B216). Both MeSH terms and related free-text terms were used. In addition, we examined the full texts of selected articles' citations to identify any missed articles. Unpublished studies and trial registries were not searched due to their lack of peer review. Two authors independently performed searches following a pre-established strategy, resolving any discrepancies through discussion.

### Selection criteria

The diagnosis of *H. pylori*-negative gastric MALT lymphoma was confirmed according to the guidelines provided by the European Society for Medical Oncology (11) and the European Gastrointestinal Lymphoma Study group (12), as well as commonly used diagnostic procedures from previous investigations. Specifically, a diagnosis of *H. pylori*-negative gastric MALT lymphoma required negative results on at least 2 tests for detecting *H. pylori*. Thus, included studies needed to meet the following criteria: (i) reporting the proportion of *H. pylori*-negative MALT lymphomas among total gastric MALT lymphomas, (ii) patients had not received prior *H. pylori* eradication therapy before the baseline of the study, and (iii) availability of full-text publications without language restrictions. Excluded studies met any of the following criteria: (i) sample size fewer than 20 patients; (ii) inclusion of non-MALT gastric lymphoma types, such as diffuse large B-cell lymphoma; (iii) use of a single or unknown method for detecting *H. pylori* infection; (iv) involvement of pediatric population in patient selection; and (v) publication types including conference abstracts, reviews, case reports, editorials, and letters. Publications of interest were selected by 2 separate authors, with translations conducted using Google Translate for non-English studies if necessary. Conflicts were resolved through discussion until a general agreement was reached.

### Data analysis

Baseline information collected for this study included the following details: first author, year of publication, country of publication, time period of research, study design, method for data collection, sample size, mean/median age of patients, male/female ratio of patients, and detection methods for *H. pylori* infection. The primary outcome was the proportion of *H. pylori*-negative gastric MALT lymphoma determined by multiple tests. Subgroup analyses were conducted to investigate the influence of different study characteristics (geographical location, number of *H. pylori* tests, publication year, and sample size) and patient characteristics (gender, primary location of lesions, number of lesions, depth of lesion invasion, and clinical stage) on the overall effect size. Secondary outcomes involved identifying distinguishing factors between *H. pylori*-negative and *H. pylori*-positive patients with gastric MALT lymphoma. When dealing with multiple publications from the same source, the most recent and/or most complete report was used for data extraction.

### Quality assessment

Article quality was assessed using the Joanna Briggs Institute Critical Appraisal Tool (13). This tool consists of a checklist with 9 items to evaluate research bias. Each item is graded as “yes,” “no,” “unclear,” or “not applicable.” A standardized quality assessment was applied to all included studies, regardless of design, as only baseline measurements were used and longitudinal data were not collected. Studies lacking information or yielding negative results on 2 items were classified as having a low risk of bias. Studies with issues in 3 items were considered to have a moderate risk of bias, while those with issues in more than 3 items were classified as having a high risk of bias (14). Quality assessments involved 2 independent authors, with inconsistencies discussed with a third author.

### Statistical analysis

The DerSimonian and Laird random-effects model was used for all analyses to address observed heterogeneities. Meta-analyses of proportions were conducted to summarize the proportion of *H. pylori*-negative gastric MALT lymphoma. The logit transformation of proportions was selected based on normality tests conducted on the original and transformed study rates (log, logit, arcsine, and double arcsine transformations). Meta-regression analyses were performed to explore study-level factors associated with the proportion of *H. pylori*-negative gastric MALT lymphoma. Potential publication bias was assessed using funnel plots of study size against log odds (15), with further testing for funnel plot asymmetry conducted using the Peter test. To determine the clinical features of *H. pylori*-negative gastric MALT lymphoma, we compared demographic and clinical characteristics of patients with gastric MALT lymphoma, both with and without *H. pylori* infection. Odds ratios (ORs) were calculated for dichotomous variables, while mean differences were calculated for continuous variables. Statistical methods suggested by Luo et al (16) were used to estimate sample mean and SD for the continuous variables represented as a median with an interquartile range. The study provided pooled estimates along with their relevant 95% confidence intervals (CIs). Statistical heterogeneity was assessed using I^2^ and Cochran Q test measures. All statistical analyses were conducted using R software (version 4.3.3, Camp Pontanezen, New Jersey).

## RESULTS

**Figure 1.**
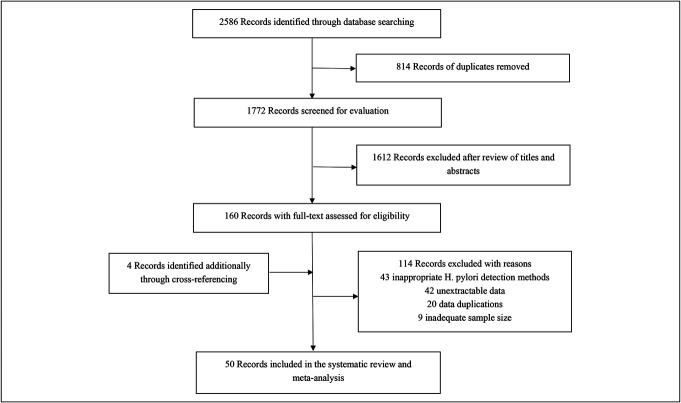
Flow diagram of the assessment of studies identified in the meta-analysis.

A total of 2,586 papers were retrieved from the initial search conducted across the Pubmed, Embase, and Cochrane Library databases. After excluding 814 duplicate articles, 1,612 were removed based on title and abstract evaluations. Following a comprehensive review of the full texts, 114 studies were excluded for various reasons (Figure 1). The category of inappropriate *H. pylori* testing methods includes studies that used only one histological or nonhistological detection method, as well as those who did not specify their detection methods. The category of unextractable data refers to studies lacking total gastric MALT lymphoma counts or those who could not differentiate MALT lymphoma data from broader gastric lymphoma data. Ultimately, 50 studies published between 1999 and 2024 met our inclusion criteria ((17–65)). Detailed characteristics of these studies are provided in Table 1. We included a total of 6,033 patients diagnosed with gastric MALT lymphomas across 12 countries/regions, predominantly in Asian countries (n = 36), with the remainder from Europe and the United States (n = 14). The risk-of-bias quality assessment for each study was performed using JBI's critical appraisal tool (see Table S2, Supplementary Digital Content, http://links.lww.com/CTG/B216). Notably, most studies did not report sample size calculation processes, resulting in an unclear rating for this aspect. The risk of bias was categorized as high in 6 studies, moderate in 13 studies, and low in 31 studies.

**Table 1.** Characteristics of included studies

Author	Year	Country/region	Time period	Study design	Data collection	Sample size (n)	Mean/median age (y)^a^	Male/female ratio (n)	Detection methods of *H. pylori*
									
Martin et al. (17)	2024	France	2000.01–2019.12	Cross-sectional study	Prospective	32	NA	12/20	Histology, PCR
Tran et al. (18)	2023	Vietnam	2020.01–2022.06	Cross-sectional study	Retrospective	22	NA	NA	Rapid urease test, urea breath test
Min et al. (19)	2023	Korea	2001.09–2020.08	Cohort study	Retrospective	203	56 (21–87)	83/120	Histology, PCR, rapid urease test, urea breath test, serology
Feng et al. (20)	2023	China	2010.01–2020.08	Cross-sectional study	Retrospective	199	NA	NA	Rapid urease test, urea breath test
Yang et al. (21)	2021	China	2003.03–2018.02	Cross-sectional study	Retrospective	70	54 (18–63)	32/38	Histology, urea breath test
Nam et al. (22)	2021	Korea	1998.01–2019.12	Cross-sectional study	Retrospective	145	53 (24–80)	80/65	Histology, urea breath test, serology
Mai et al. (23)	2021	America	2004.06–2019.06	Cross-sectional study	Retrospective	56	58 (21–89)	27/29	Histology, urea breath test, stool antigen, serology
Kim et al. (24)	2021	Korea	2000.01–2018.12	Cohort study	Retrospective	1,163	56 ± 12	493/670	Histology, rapid urease test, urea breath test
Wu et al. (25)	2020	China	2013.01–2018.12	Cross-sectional study	Retrospective	33	54.1 ± 11.6	18/15	Histology, urea breath test
Puig et al. (26)	2020	Spain	1998.01–2009.01	Cross-sectional study	Retrospective	198	62.8 ± 13.0	88/110	Histology, culture, rapid urease test, urea breath test, stool antigen, serology
Kiesewetter et al. (27)	2020	Austria	1999–2019	Cohort study	Retrospective	137	63 (22–85)	70/67	Histology, serology
Matysiak-Budnik et al. (28)	2019	France	2002–2010	Cross-sectional study	Retrospective	416	67 (23–97)	209/207	Histology, urea breath test, serology
Song et al. (29)	2018	China	2000.01–2013.01	Cross-sectional study	Retrospective	122	56.5 (19–84)	66/56	Histology, rapid urease test, urea breath test
Rentien et al. (30)	2018	France	1995.01–2014.01	Cohort study	Retrospective	179	57.4 ± 13.7	94/85	Histology, PCR
Choi et al. (31)	2018	Korea	2001.01–2016.12	Cross-sectional study	Retrospective	132	52 (17–81)	47/85	Histology, rapid urease test, urea breath test, serology
Park et al. (32)	2017	Korea	2010–2012	Cross-sectional study	Retrospective	52	52.4 (23–80)	21/31	Histology, rapid urease test, urea breath test, serology
Kuo et al. (33)	2017	Taiwan	2005.01–2014.06	Cross-sectional study	Retrospective	93	NA	NA	Histology, rapid urease test, urea breath test, serology
Iwamuro et al. (34)	2017	Japan	1997.10–2015.11	Cross-sectional study	Retrospective	146	65.4 ± 12.6	70/76	Histology, rapid urease test, urea breath test, stool antigen, serology
Moleiro et al. (35)	2016	Portugal	1993–2013	Cross-sectional study	Retrospective	144	56 ± 13	76/68	Histology, culture, urea breath test
Li et al. (36)	2016	China	2001.08–2013.08	Cross-sectional study	Retrospective	103	53 (19–85)	52/51	Histology, urea breath test
Kim et al. (37)	2016	Korea	2001.01–2014.06	Cohort study	Retrospective	54	58.8 ± 9.8	22/32	Histology, rapid urease test, urea breath test
Gong et al. (38)	2016	Korea	1995.11–2014.09	Cohort study	Retrospective	345	53 (45–60)	150/195	Histology, rapid urease test, urea breath test, serology
Tajika et al. (39)	2014	Japan	1994.03–2011.06	Cohort study	Prospective	146	59 (26–87)	68/78	Histology, culture, rapid urease test, urea breath test, serology
Ryu et al. (40)	2014	Korea	2000.12–2012.06	Cohort study	Retrospective	57	NA	24/33	Histology, rapid urease test, urea breath test, serology
Nam et al. (41)	2014	Korea	2000.01–2012.09	Cross-sectional study	Retrospective	48	53 (25–85)	25/23	Histology, rapid urease test, urea breath test, serology
Min et al. (42)	2014	Korea	2000.01–2010.12	Cross-sectional study	Retrospective	194	53.3 ± 11.4	88/106	Histology, rapid urease test, urea breath test, serology
Choi et al. (43)	2013	Korea	2003.05–2012.04	Cohort study	Retrospective	66	53.8 ± 11.2	29/37	Histology, rapid urease test, urea breath test, serology
Asano et al. (44)	2012	Japan	1995.04–2012.05	Cross-sectional study	Retrospective	158	NA	NA	Histology, rapid urease test, urea breath test, serology
Sumida et al. (45)	2009	Japan	1997–2007	Cross-sectional study	Retrospective	66	59.8 (20–89)	29/37	Histology, rapid urease test, urea breath test, serology
Stathis et al. (46)	2009	Switzerland	1990.07–2006.11	Cross-sectional study	Retrospective	105	64 (20–94)	51/54	Histology, urea breath test, serology
Chung et al. (24)	2009	Korea	1996–2006	Cohort study	Retrospective	185	NA	87/98	Histology, rapid urease test
Yamamoto et al. (47)	2008	Japan	1993.10–2005.12	Cohort study	Retrospective	67	NA	29/38	Histology, culture, rapid urease test, urea breath test, serology
Todorovic et al. (48)	2008	Serbia	1997.07–2003.09	Cross-sectional study	Retrospective	34	62 (34–82)	18/16	Histology, culture
Nakamura et al. (49)	2008	Japan	1993.11–2006.09	Cohort study	Retrospective	86	56.6 (26–87)	39/47	Histology, culture, rapid urease test, urea breath test, serology
Dong et al. (50)	2008	China	2001–2007	Cross-sectional study	Retrospective	22	58 (18–78)	10/12	Histology, urea breath test, serology
Nakamura et al. (51)	2006	Japan	1993–2005	Cross-sectional study	Retrospective	110	62 (16–84)	49/61	Histology, culture, rapid urease test, urea breath test, serology
Gisbert et al. (52)	2006	Spain	1991.01–2005.12	Cross-sectional study	Retrospective	37	61 ± 14	23/15	Histology, urea breath test
Cheng et al. (53)	2006	Taiwan	1998–2005	Cross-sectional study	Retrospective	62	56.2 ± 13.4	32/30	Histology, culture, rapid urease test, serology
Akamatsu et al. (54)	2006	Japan	1993–2006	Cross-sectional study	Retrospective	57	NA	29/28	Histology, culture, urea breath test, serology
Lévy et al. (55)	2005	France	1995–2005	Cohort study	Retrospective	53	54.2 (29–74)	31/22	Histology, culture
Chen et al. (56)	2005	Taiwan	1995.06–2004.01	Cohort study	Prospective	34	60 (30–84)	15/19	Histology, culture, rapid urease test
Bao et al. (57)	2005	China	1999.01–2005.03	Cross-sectional study	Retrospective	22	60.9 (26–78)	10/12	Histology, PCR, rapid urease test
Lee et al. (58)	2004	Korea	1992.05–2002.08	Cross-sectional study	Retrospective	55	47.8 ± 11.3	24/31	Histology, rapid urease test
Iwano et al. (59)	2004	Japan	1995–2002	Cross-sectional study	Retrospective	63	NA	NA	Histology, culture, rapid urease test, urea breath test, serology
Inagaki et al. (60)	2004	Japan	NA	Cross-sectional study	Retrospective	115	59 (16–87)	65/50	Histology, culture, rapid urease test, urea breath test, serology
Yeh et al. (61)	2003	Taiwan	1994.01–2002.03	Cross-sectional study	Retrospective	20	61 ± 15	11/9	Histology, culture, urea breath test
Lehours et al. (62)	2003	France	1995–2000	Cohort study	Prospective	90	56.3 (21–76)	53/37	Histology, culture, PCR, serology
Goda et al. (63)	2003	Japan	1993.08–2002.03	Cross-sectional study	Retrospective	24	61 (45–83)	7/17	Histology, culture, rapid urease test, urea breath test, serology
Weston et al. (64)	1999	America	1993–1997	Cross-sectional study	Prospective	68	61.8 ± 13.5	67/1	Histology, serology
Steinbach et al. (65)	1999	America	NA	Cohort study	Prospective	34	57 (26–77)	15/19	Histology, rapid urease test, serology

*H. pylori*, *Helicobacter pylori;*PCR, polymerase chain reaction.

a The age distribution of included patients is expressed as mean ± SD or median (interquartile range/min-max range).

### Proportion of *H. pylori*-negative gastric MALT lymphoma overall and by study or patient characteristics

**Figure 2.**
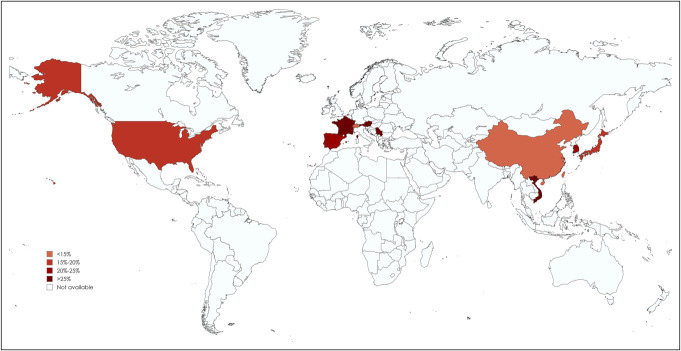
Proportion of *H. pylori*-negative gastric mucosa-associated lymphoid tissue lymphoma worldwide.

The overall proportion of *H. pylori*-negative gastric MALT lymphoma was estimated to be 20.5% (95% CI 17.0%–24.6%) (Table 2, Figure 2), with significant heterogeneity observed among studies (I^2^ = 89%). On subgroup analysis by study characteristics, the regional proportion was 18.1% in Asian countries (95% CI 14.8%–21.8%, I^2^ = 87%) and 28.3% in non-Asian countries (95% CI 19.3%–39.4%, I^2^ = 88%). Further analysis revealed similar rates between studies using ≤3 tests for *H. pylori* detection (20.5%; 95% CI 15.1%–27.2%, I^2^ = 90%) and those using >3 tests (20.4%; 95% CI 16.2%–25.3%, I^2^ = 86%). A significantly lower pooled proportion was observed for publications in or before 2013 (15.0% vs. 26.8%) and those with sample sizes ≤100 (19.5% vs. 21.7%). Additional analyses based on different countries/regions, World Bank income levels, and different *H. pylori* detection tests are available in Tables S3-S4 (see Supplementary Digital Content, http://links.lww.com/CTG/B216).

**Table 2.** Pooled proportion of *H. pylori*-negative gastric MALT lymphoma

Population	Studies (n)	Patients (n)	Prevalence (95% CI)	I^2^ (%)	*P* value
Overall	50	6,033	0.205 (0.170–0.246)	89	<0.01
Subgroup					
Country of origin					
Asian	36	4,508	0.181 (0.148–0.218)	87	<0.01
Non-Asian	14	1,525	0.283 (0.193–0.394)	88	<0.01
*H. pylori* detection					
≤ 3 tests	26	3,433	0.205 (0.151–0.272)	90	<0.01
> 3 tests	24	2,600	0.204 (0.162–0.253)	86	<0.01
Publication yr					
≤ 2013	24	1,562	0.150 (0.121–0.184)	56	<0.01
>2013	26	4,471	0.268 (0.211–0.333)	92	<0.01
Sample size					
≤100 patients	29	1,441	0.195 (0.149–0.251)	77	<0.01
>100 patients	21	4,592	0.217 (0.166–0.279)	94	<0.01
Gender					
Female	10	647	0.149 (0.096–0.226)	76	<0.01
Male	10	519	0.250 (0.153–0.380)	79	<0.01
Dominant site					
Distal	7	309	0.202 (0.125–0.309)	71	<0.01
Proximal	7	268	0.218 (0.095–0.427)	78	<0.01
No. of lesions					
Single	3	72	0.167 (0.097–0.271)	0	0.43
Multiple	3	105	0.217 (0.117–0.365)	66	0.05
Endoscopic morphology					
Superficial type	4	185	0.189 (0.136–0.255)	25	0.26
Nonsuperficial type	4	58	0.224 (0.135–0.349)	0	0.61
Depth of invasion					
Mucosa	4	324	0.074 (0.050–0.108)	2	0.38
Submucosa or beyond	4	177	0.243 (0.077–0.551)	77	<0.01
Clinical stage (Lugano staging)					
Stage I	5	424	0.166 (0.129–0.211)	20	0.29
Stage II or more	5	57	0.432 (0.191–0.709)	68	0.01
Clinical stage (modified Ann Arbor staging)					
Stage IE	5	1,692	0.159 (0.106–0.232)	90	<0.01
Stage IIE	5	105	0.314 (0.233–0.409)	0	0.97
Stage IIIE/IV	4	126	0.397 (0.274–0.536)	51	0.11

CI, confidence interval; *H. pylori*, *Helicobacter pylori*; MALT, mucosa-associated lymphoid tissue.

On subgroup analysis by patient characteristics, the proportion of *H. pylori*-negative gastric MALT lymphoma was 14.9% (95% CI 9.6%–22.6%, I^2^ = 76%) in female patients and 25.0% (95% CI 15.3%–38.0%, I^2^ = 79%) in male patients. Lower rates were observed in patients with distal lesions (20.2%; 95% CI 12.5%–30.9%, I^2^ = 71%) compared with proximal lesions (21.8%; 95% CI 9.5%–42.7%, I^2^ = 78%), in single lesions (16.7%; 95% CI 9.7%–27.1%, I^2^ = 0%) compared with multiple lesions (21.7%; 95% CI 11.7%–36.5%, I^2^ = 66%), and in superficial type morphology (18.9%; 95% CI 13.6%–25.5%, I^2^ = 25%) compared with nonsuperficial type morphology (22.4%; 95% CI 13.5%–34.9%, I^2^ = 0%). A stark contrast was noted between patients with mucosal invasion (7.4%; 95% CI 5.0%–10.8%, I^2^ = 2%) and those with submucosal (or beyond) invasion (24.3%; 95% CI 7.7%–55.1%, I^2^ = 77%). The proportion of *H. pylori*-negative gastric MALT lymphoma also increased with the clinical stage: 16.6% (95% CI 12.9%–21.2%, I^2^ = 20%) in Lugano stage I and 43.2% (95% CI 19.2%–70.9%, I^2^ = 68%) in Lugano stage II or higher. Using modified Ann Arbor staging, the proportions were 15.9% (95% CI 10.6%–23.2%, I^2^ = 90%) in stage IE, 31.4% (95% CI 23.3%–40.9%, I^2^ = 0%) in stage IIE, and 39.7% (95% CI 27.4%–53.6%, I^2^ = 51%) in stage IIIE/IV. Forest plots for these proportions are presented in Figures S1-24 (See Supplementary Digital Content, http://links.lww.com/CTG/B216).

### Association of baseline characteristics with *H. pylori*-negative gastric MALT lymphoma

Data from 10 studies compared the characteristics of gastric MALT lymphoma patients with and without *H. pylori* infection (Table 3). We found that *H. pylori*-negative patients with gastric MALT lymphoma were more likely to be male (OR 1.858; 95% CI 1.282–2.691, I^2^ = 16%), have proximal lesions (OR 1.777; 95% CI 1.028–3.070, I^2^ = 2%), and exhibit submucosal (or beyond) invasion (OR 4.028; 95% CI 1.078–15.046, I^2^ = 64%). These patients also tended to present with clinical stages of Lugano stage II or higher (OR 3.895; 95% CI 1.574–9.636, I^2^ = 30%), modified Ann Arbor stage IIE (OR 1.604; 95% CI 1.028–2.505, I^2^ = 0%), or modified Ann Arbor stage IIIE/IV (OR 3.292; 95% CI 2.237–4.844, I^2^ = 0%). Conversely, they were less likely to present with Lugano stage I (OR 0.257; 95% CI 0.104–0.635, I^2^ = 30%) or modified Ann Arbor stage IE (OR 0.372; 95% CI 0.268–0.518, I^2^ = 0%). No significant differences were observed between groups regarding patient age, number of lesions, superficial type morphology, or presence of t(11; 18) (q21; q21) translocation. Forest plots depicting these associations are shown in Figures S25-36 (see Supplementary Digital Content, http://links.lww.com/CTG/B216).

**Table 3.** Association of basic characteristics with *H. pylori*-negative gastric MALT lymphoma

Characteristics	Studies (n)	Patients (n)		Effect size^a^ (95% CI)	I^2^ (%)	*P* value
		HP (−) MALT	HP (+) MALT			
Age (yr)	8	170	873	1.577 (−0.248 to 3.403)	14	0.090
Gender, male	10	193	973	1.858 (1.282–2.691)	16	0.001
Location, proximal	7	111	459	1.777 (1.028–3.070)	2	0.040
Lesions, multiple	3	35	142	1.401 (0.340–5.770)	65	0.640
Morphology, superficial type	4	48	195	0.681 (0.294–1.578)	10	0.371
Invasion, submucosal or beyond	4	47	468	4.028 (1.078–15.046)	64	0.038
Positivity, t(11; 18) (q21; q21)	4	45	108	3.803 (0.919–15.737)	62	0.065
Clinical stage, Lugano stage I	5	89	392	0.257 (0.104–0.635)	30	0.003
Clinical stage, Lugano stage II or more	5	89	392	3.895 (1.574–9.636)	30	0.003
Clinical stage, Ann Arbor stage IE	5	407	1,516	0.372 (0.268–0.518)	0	<0.001
Clinical stage, Ann Arbor stage IIE	5	407	1,516	1.604 (1.028–2.505)	0	0.038
Clinical stage, Ann Arbor stage IIIE/IV	4	391	1,475	3.292 (2.237–4.844)	0	<0.001

CI, confidence interval; *H. pylori*/HP, *Helicobacter pylori*; MALT, mucosa-associated lymphoid tissue.

a The odds ratio (OR) and mean difference (MD) were used as effect measures for dichotomous and continuous variables, respectively.

### Heterogeneity, temporal trend, and publication bias

Univariable meta-regression analysis indicated that country of origin (*P* = 0.011) and publication year (*P* = 0.002) significantly correlated with the proportion rate. Multivariate analysis confirmed that these factors collectively accounted for 36% of the observed heterogeneity (see Table S5, Supplementary Digital Content, http://links.lww.com/CTG/B216). Meta-regression was also used to explore the temporal trends, revealing an increasing pattern in the proportion of *H. pylori*-negative gastric MALT lymphoma over time (coefficient 0.058; 95% CI 0.028–0.089, *P* = 0.0003; see Figure S37, Supplementary Digital Content, http://links.lww.com/CTG/B216). Analysis of each study's end point time as an independent variable demonstrated a simultaneous rise in proportions (coefficient 0.064; 95% CI: 0.027–0.100, *P* = 0.0009; see Figure S38, Supplementary Digital Content, http://links.lww.com/CTG/B216). No significant asymmetry was detected in the funnel plot for the primary outcome (see Figures S39, Supplementary Digital Content, http://links.lww.com/CTG/B216), and the Peter test did not indicate any apparent publication bias (*P* = 0.510).

## DISCUSSION

To the best of our knowledge, this is the first systematic review and meta-analysis reporting the proportion and characteristics of patients with gastric MALT lymphoma who tested negative for *H. pylori*. Our study reveals a notable incidence of *H. pylori*-negative cases, which appears to be increasing annually. Clinically, these patients often demonstrate deeper invasion and more advanced stages compared with their *H. pylori*-positive counterparts. Given the declining occurrence of *H. pylori*-positive cases in recent years (66,67), these findings highlight the need to enhance clinical focus on this specific patient population.

Our study indicates that over 20% of patients with gastric MALT lymphoma are *H. pylori*-negative, as determined by 2 or more negative tests. Surprisingly, the number of *H. pylori* tests did not significantly affect the proportion of *H. pylori*-negative gastric MALT lymphoma (as indicated by the pooled rate and meta-regression results). This suggests that 2 *H. pylori* tests have effectively reduced false-negative results already. Furthermore, our findings show that this patient group is less common in Asian countries compared with non-Asian countries, possibly due to a higher rate of *H. pylori* infection in Asia (68). A higher proportion was also observed in high-income countries (see Table S3, Supplementary Digital Content, http://links.lww.com/CTG/B216), further suggesting the influence of social-environmental factors on this rate. Regarding patient characteristics, male gender, proximal or multiple lesions, nonsuperficial appearance during endoscopy, and invasion into the submucosal layer were associated with a higher proportion of *H. pylori*-negative gastric MALT lymphoma. This indicates variation among different subpopulations and suggests distinct clinical characteristics for this type of MALT lymphoma. In addition, our findings indicate that this rate increases with the clinical stage of patients, suggesting that *H. pylori*-negative patients may have a poorer prognosis (24). Ultimately, we discovered a rising trend over time in the occurrence of *H. pylori*-negative gastric MALT lymphoma, as indicated by both publication and inclusion dates. This trend may be attributed to increased antibiotic use, leading to a decline in the proportion of *H. pylori* infections (69). It is also possible that while the *H. pylori* infection rate is declining, other pathogens or antigens are playing a greater role in gastric pathology (25).

In terms of clinical characteristics, *H. pylori*-negative gastric MALT lymphoma occurs more frequently in male patients. The reason for this remains uncertain; however, a previous study using extensive urea breath tests indicated that women may have a higher bacterial load (70). Therefore, variations in the interactions between *H. pylori* and the host may account for this inconsistency (71). Furthermore, our study revealed that gastric MALT lymphoma without *H. pylori* infection is more likely to develop in the proximal stomach, possibly due to higher concentrations of *H. pylori* organisms and acquired lymphoid tissue in the distal portion (65,72). In addition, we found that gastric MALT lymphoma without *H. pylori* infection has a greater susceptibility to submucosal invasion, which previous studies have linked to a suboptimal response to eradication therapy (73). This also partially explains why less than one-third of patients with *H. pylori*-negative gastric MALT achieve clinical remission through *H. pylori* eradication (74). In this study, we did not observe a higher frequency of t(11; 18) (q21; q21) translocation in *H. pylori*-negative gastric MALT lymphomas. However, the borderline statistical result (*P* = 0.065) suggests that this may be due to the limited sample size included. The t(11; 18) (q21; q21) translocation also significantly contributes to *H. pylori* resistance to eradication therapy (75). Ultimately, we found that this specific group of patients often presents with a more advanced clinical stage compared with *H. pylori*-positive patients, as supported by both the Lugano grading (76) and the modified Ann Arbor grading (77). Given the differences in treatment recommendations between current ESMO (8) and National Comprehensive Cancer Network guidelines (9) for this particular patient group, our study indicates the need for additional patient classification, and prompt initiation of targeted treatment (often radiotherapy) may be necessary (24,78).

This study has several limitations. First, significant heterogeneity in proportion estimates was observed. We conducted meta-regression models to identify the source of this heterogeneity by analyzing study-level characteristics. However, factors at the patient level, such as gender, lesion location and number, lesion appearance during endoscopy, invasion extent, and clinical stage, may also contribute to the heterogeneity. Second, some outcomes should be interpreted with caution. The precise definition of distal or proximal lesions is not clearly described in some studies, which may lead to inaccuracies in meta-analysis results. We also found significant interstudy variation in the morphological categorization of endoscopic observations. While studies with similar classifications were pooled, 2 studies were excluded from the analysis due to distinct classification criteria. Third, the treatment outcomes for *H. pylori*-negative gastric MALT lymphoma were not explored. Although treatment methods and patient prognosis are clinical focuses, we did not analyze them, as prior meta-analyses have already investigated the efficacy of eradication therapy for individuals without *H. pylori* infection (74,79). In addition, there is currently a scarcity of well-conducted randomized controlled trials to provide further clarity on appropriate treatment approaches for this specific population. Finally, while we identified several correlations between baseline characteristics and *H. pylori*-negative gastric MALT lymphoma, it is important to note that some characteristics such as patients' comorbidities and lifestyle habits have not been reported in the included studies. The causal relationship between all these factors and *H. pylori*-negative gastric MALT lymphoma still requires confirmation through prospective longitudinal studies.

In conclusion, our study provides a comprehensive analysis of the proportion and associated characteristics of *H. pylori*-negative gastric MALT lymphoma. Further research is essential to determine optimal treatment and surveillance strategies, given its potential for increased incidence and advanced clinical features.

## CONFLICTS OF INTEREST

**Guarantor of the article:** Jin-Lin Yang, MD.

**Specific author contributions:** X.-H.L., Z.W., and J.-L.Y.: conceived the study. X.-H.L., Q.L., J.-H.L., and B.H.X.: conducted the literature search, screened the studies, extracted the data, and discussed the results with Z.-J.W. X.-H.L. and Q.L.: conducted the meta-analysis and drafted the manuscript. Z.W. and J.-L.Y.: supervised the manuscript. All the authors have read and approved the final manuscript, including the authorship list.

**Financial support:** This study is funded by the National Natural Science Foundation of China (Grant Nos. 82173253, 82103539).

**Potential competing interests:** None to report.

## Supplementary Material
