# Supplementary material for: Proportion and Characteristics of Helicobacter Pylori-Negative Gastric Mucosa-Associated Lymphoid Tissue Lymphoma: A Systematic Review and Meta-Analysis
Source: Clin Transl Gastroenterol. 2024 Oct 25;16(4):e00781. doi: 10.14309/ctg.0000000000000781 (PMC12020693; doi:10.14309/ctg.0000000000000781)
Supplement: Supplementary file 1 [file ct9-16-e00781-s001.docx]

**Proportion and characteristics of Helicobacter pylori-negative gastric mucosa-associated lymphoid tissue lymphoma: A systematic review and meta-analysis**

**Contents**

[**SEARCH STRATEGY** 4](#_Toc178080749)

[Table S1. Detailed search strategy. 4](#_Toc178080750)

[**QUALITY ASSESSMENT** 7](#_Toc178080751)

[Table S2. Quality assessment of included studies. 7](#_Toc178080752)

[**proportion of Helicobacter pylori-negative gastric mucosa-associated lymphoid tissue lymphoma** 11](#_Toc178080753)

[Figure S1. Proportion of H. pylori-negative gastric MALT lymphoma of all included studies 11](#_Toc178080754)

[Figure S2. Proportion of H. pylori-negative gastric MALT lymphoma in studies published in Asian countries 12](#_Toc178080755)

[Figure S3. Proportion of H. pylori-negative gastric MALT lymphoma in studies published in non-Asian countries 12](#_Toc178080756)

[Figure S4. Proportion of H. pylori-negative gastric MALT lymphoma in studies applying ≤ 3 H. pylori detection tests 13](#_Toc178080757)

[Figure S5. Proportion of H. pylori-negative gastric MALT lymphoma in studies applying > 3 H. pylori detection tests 13](#_Toc178080758)

[Figure S6. Proportion of H. pylori-negative gastric MALT lymphoma in studies with a publication year ≤ 2013 14](#_Toc178080759)

[Figure S7. Proportion of H. pylori-negative gastric MALT lymphoma in studies with a publication year > 2013 14](#_Toc178080760)

[Figure S8. Proportion of H. pylori-negative gastric MALT lymphoma in studies with a sample size ≤ 100 participants 15](#_Toc178080761)

[Figure S9. Proportion of H. pylori-negative gastric MALT lymphoma in studies with a sample size >100 participants 15](#_Toc178080762)

[Figure S10. Proportion of H. pylori-negative gastric MALT lymphoma in male patients 16](#_Toc178080763)

[Figure S11. Proportion of H. pylori-negative gastric MALT lymphoma in female patients 16](#_Toc178080764)

[Figure S12. Proportion of H. pylori-negative gastric MALT lymphoma in patients with distal lesions 17](#_Toc178080765)

[Figure S13. Proportion of H. pylori-negative gastric MALT lymphoma in patients with proximal lesions 17](#_Toc178080766)

[Figure S14. Proportion of H. pylori-negative gastric MALT lymphoma in patients with single lesion 18](#_Toc178080767)

[Figure S15. Proportion of H. pylori-negative gastric MALT lymphoma in patients with multiple lesions 18](#_Toc178080768)

[Figure S16. Proportion of H. pylori-negative gastric MALT lymphoma in patients with endoscopic morphology of superficial type 19](#_Toc178080769)

[Figure S17. Proportion of H. pylori-negative gastric MALT lymphoma in patients with endoscopic morphology of non-superficial type 19](#_Toc178080770)

[Figure S18. Proportion of H. pylori-negative gastric MALT lymphoma in patients with mucosal invasion 20](#_Toc178080771)

[Figure S19. Proportion of H. pylori-negative gastric MALT lymphoma in patients with submucosal (or beyond) invasion 20](#_Toc178080772)

[Figure S20. Proportion of H. pylori-negative gastric MALT lymphoma in patients with Lugano stage I 21](#_Toc178080773)

[Figure S21. Proportion of H. pylori-negative gastric MALT lymphoma in patients with Lugano stage II or more 21](#_Toc178080774)

[Figure S22. Proportion of H. pylori-negative gastric MALT lymphoma in patients with modified Ann Arbor stage IE 22](#_Toc178080775)

[Figure S23. Proportion of H. pylori-negative gastric MALT lymphoma in patients with modified Ann Arbor stage IIE 22](#_Toc178080776)

[Figure S24. Proportion of H. pylori-negative gastric MALT lymphoma in patients with modified Ann Arbor stage IIIE/IV 22](#_Toc178080777)

[Table S3. Pooled proportion of H. pylori-negative gastric MALT lymphoma based on different countries/regions and relevant World Bank income level 23](#_Toc178080778)

[Table S4. Pooled proportion of H. pylori-negative gastric MALT lymphoma based on different diagnostic tests for H. pylori 24](#_Toc178080779)

[**CHARACTERISTICS OF HELICOBACTER PYLORI-NEGATIVE GASTRIC MUCOSA-ASSOCIATED LYMPHOID TISSUE LYMPHOMA** 26](#_Toc178080780)

[Figure S25. Association of patients’age with H. pylori-negative gastric MALT lymphoma 26](#_Toc178080781)

[Figure S26. Association of male gender with H. pylori-negative gastric MALT lymphoma 26](#_Toc178080782)

[Figure S27. Association of proximal lesions with H. pylori-negative gastric MALT lymphoma 26](#_Toc178080783)

[Figure S28. Association of multiple lesions with H. pylori-negative gastric MALT lymphoma 27](#_Toc178080784)

[Figure S29. Association of superficial type morphology with H. pylori-negative gastric MALT lymphoma 27](#_Toc178080785)

[Figure S30. Association of submucosal (or beyond) invastion with H. pylori-negative gastric MALT lymphoma 27](#_Toc178080786)

[Figure S31. Association of t(11;18)(q21;q21) positivity with H. pylori-negative gastric MALT lymphoma 27](#_Toc178080787)

[Figure S32. Association of clinical stage (Lugano stage I) with H. pylori-negative gastric MALT lymphoma 28](#_Toc178080788)

[Figure S33. Association of clinical stage (Lugano stage II or more) with H. pylori-negative gastric MALT lymphoma 28](#_Toc178080789)

[Figure S34. Association of clinical stage (modified Ann Arbor stage IE) with H. pylori-negative gastric MALT lymphoma 29](#_Toc178080790)

[Figure S35. Association of clinical stage (modified Ann Arbor stage IIE) with H. pylori-negative gastric MALT lymphoma 29](#_Toc178080791)

[Figure S36. Association of clinical stage (modified Ann Arbor stage IIIE/IV) with H. pylori-negative gastric MALT lymphoma 29](#_Toc178080792)

[**HETEROGENEITY, TEMPORAL TREND, AND PUBLICATION BIAS** 30](#_Toc178080793)

[Table S5. Meta-regression analysis on the proportion of H. pylori-negative gastric MALT lymphoma 30](#_Toc178080794)

[Figure S37. Temporal trend for the proportion of H. pylori-negative gastric MALT lymphoma based on publication year 31](#_Toc178080795)

[Figure S38. Temporal trend for the proportion of H. pylori-negative gastric MALT lymphoma based on inclusion year 31](#_Toc178080796)

[Figure S39. Funnel plot for the proportion of H. pylori-negative gastric MALT lymphoma 32](#_Toc178080797)

**SEARCH STRATEGY**

**Table S1. Detailed search strategy.**

| Pubmed (2024-03-26) | | |
| --- | --- | --- |
| Number | Items | Results |
| #1 | "Lymphoma, B cell, Marginal Zone"[MeSH Terms] | 5499 |
| #2 | "mucosa-associated lymphoid tissue lymphoma"[Title/Abstract] OR "mucosa associated lymphoid tissue lymphoma"[Title/Abstract] OR "b-cell lymphoma"[Title/Abstract] OR "b cell lymphoma"[Title/Abstract] OR "marginal-zone lymphoma"[Title/Abstract] OR "marginal zone lymphoma"[Title/Abstract] OR "MALT"[Title/Abstract] OR "MALToma"[Title/Abstract] | 47059 |
| #3 | #1 OR #2 | 47840 |
| #4 | "gastric"[Title/Abstract] OR "stomach"[Title/Abstract] | 380147 |
| #5 | #3 AND #4 | 3856 |
| #6 | "Helicobacter pylori"[MeSH Terms] | 39019 |
| #7 | "Helicobacter pylori"[Title/Abstract] OR "H. pylori"[Title/Abstract] "H pylori"[Title/Abstract] OR "Campylobacter pylori*"[Title/Abstract] OR "Helicobacter nemestrinae"[Title/Abstract] OR "Hp"[Title/Abstract] | 60483 |
| #8 | #6 OR #7 | 72108 |
| #9 | #5 AND #8 | 1787 |
| #10 | #9 NOT ((case reports[Publication Type]) OR (comment[Publication Type]) OR (editorial[Publication Type]) OR (letter[Publication Type]) OR (review[Publication Type]) OR (guideline[Publication Type])) | 886 |
|  | | |
| Embase (2024-03-26) | | |
| Number | Items | Results |
| #1 | 'malt lymphoma'/exp | 410 |
| #2 | 'mucosa-associated lymphoid tissue lymphoma':ab,kw,ti OR 'mucosa associated lymphoid tissue lymphoma':ab,kw,ti OR 'b-cell lymphoma':ab,kw,ti OR 'b cell lymphoma':ab,kw,ti OR 'marginal-zone lymphoma':ab,kw,ti OR 'marginal zone lymphoma':ab,kw,ti OR 'MALT':ab,kw,ti OR 'MALToma':ab,kw,ti | 75408 |
| #3 | #1 OR #2 | 75464 |
| #4 | 'gastric':ab,kw,ti OR 'stomach':ab,kw,ti | 522645 |
| #5 | #3 AND #4 | 5868 |
| #6 | 'helicobacter pylori'/exp | 63371 |
| #7 | 'Helicobacter pylori':ab,kw,ti OR 'H. pylori':ab,kw,ti OR 'H pylori':ab,kw,ti OR 'Campylobacter pylori*':ab,kw,ti OR 'Helicobacter nemestrinae':ab,kw,ti OR 'Hp':ab,kw,ti | 106762 |
| #8 | #6 OR #7 | 117583 |
| #9 | #5 AND #8 | 3031 |
| #10 | #9 NOT ('review'/it OR 'conference review'/it OR 'editorial'/it OR 'letter'/it OR 'note'/it OR 'erratum'/it) | 1596 |
|  | | |
| Cochrane Library (2024-03-26) | | |
| Number | Items | Results |
| #1 | MeSH descriptor: [Lymphoma, B cell, Marginal Zone] explode all trees | 82 |
| #2 | (mucosa-associated lymphoid tissue lymphoma):ab,kw,ti OR (mucosa associated lymphoid tissue lymphoma):ab,kw,ti OR (b-cell lymphoma):ab,kw,ti OR (b cell lymphoma):ab,kw,ti OR (marginal-zone lymphoma):ab,kw,ti OR (marginal zone lymphoma):ab,kw,ti OR (MALT):ab,kw,ti OR (MALToma):ab,kw,ti | 4756 |
| #3 | #1 OR #2 | 4756 |
| #4 | (gastric):ab,kw,ti OR (stomach):ab,kw,ti | 40334 |
| #5 | #3 AND #4 | 179 |
| #6 | MeSH descriptor: [Helicobacter pylori] explode all trees | 2569 |
| #7 | (Helicobacter pylori):ab,kw,ti OR (H. pylori):ab,kw,ti OR (H pylori):ab,kw,ti OR (Campylobacter pylori*):ab,kw,ti OR (Helicobacter nemestrinae):ab,kw,ti OR (Hp):ab,kw,ti | 9262 |
| #8 | #6 OR #7 | 9262 |
| #9 | #5 AND #8 | 104 |
| #10 | #9 limited to Trials | 104 |

**QUALITY ASSESSMENT**

**Table S2. Quality assessment of included studies.**

| Study | Sample  frame^1#^ | Sampling  method^2#^ | Sample  Size^3#^ | Subjects  and setting^4#^ | Sample  Coverage^5#^ | Identification  of condition^6#^ | Measurement  of condition^7#^ | Statistical  Analysis^8#^ | Response  Rate^9#^ |
| --- | --- | --- | --- | --- | --- | --- | --- | --- | --- |
| Martin A 2024 | Yes | Yes | Unclear | Yes | Yes | Yes | Yes | Yes | NA |
| Tran Q 2023 | Yes | Yes | Unclear | Yes | Yes | Yes | Yes | Yes | NA |
| Min G 2023 | Yes | Yes | Unclear | Yes | Yes | Yes | Yes | Yes | Yes |
| Feng Y 2023 | Yes | Yes | Unclear | Yes | Yes | Yes | Yes | Yes | NA |
| Yang H 2021 | Yes | Yes | Unclear | Yes | No | Yes | Yes | No | NA |
| Nam H 2021 | Yes | Yes | Unclear | Yes | Yes | Yes | Yes | Yes | Yes |
| Mai B 2021 | Yes | Yes | Unclear | Yes | No | Yes | Yes | Yes | NA |
| Kim J 2021 | Yes | Yes | Yes | Yes | Yes | Yes | Yes | Yes | Yes |
| Wu Y 2020 | Yes | Yes | Unclear | Yes | Yes | Yes | Yes | Yes | NA |
| Puig I 2020 | Yes | Yes | Unclear | Yes | No | Yes | Yes | Yes | NA |
| Kiesewetter B 2020 | Yes | Yes | Unclear | Yes | Yes | Yes | Yes | Yes | Yes |
| Matysiak-Budnik T 2019 | Yes | Yes | Yes | Yes | No | Yes | Yes | Yes | Yes |
| Song Y 2018 | Yes | Yes | Unclear | Yes | No | Yes | Yes | Yes | NA |
| Rentien A 2018 | Yes | Yes | Unclear | Yes | Yes | Yes | Yes | Yes | Yes |
| Choi S 2018 | Yes | Yes | Unclear | Yes | No | Yes | Yes | Yes | NA |
| Park S 2017 | Yes | Yes | Unclear | Yes | No | Yes | Yes | Yes | NA |
| Kuo S 2017 | Yes | Yes | Unclear | Yes | No | Yes | Yes | Yes | NA |
| Iwamuro M 2017 | Yes | No | Unclear | Yes | Yes | Yes | Yes | Yes | NA |
| Moleiro J 2016 | Yes | Yes | Unclear | Yes | Yes | Yes | Yes | Yes | Yes |
| Li X 2016 | Yes | Yes | Unclear | Yes | Yes | Yes | Yes | Yes | Yes |
| Kim J 2016 | Yes | Yes | Unclear | Yes | Yes | Yes | Yes | Yes | NA |
| Gong E 2016 | Yes | Yes | Unclear | Yes | Yes | Yes | Yes | Yes | NA |
| Tajika M 2014 | Yes | Yes | Unclear | Yes | Yes | Yes | Yes | Yes | NA |
| Ryu K 2014 | Yes | Yes | Unclear | Yes | Yes | Yes | Yes | Yes | NA |
| Nam T 2014 | Yes | Yes | Unclear | Yes | No | Yes | Yes | Yes | NA |
| Min B 2014 | Yes | Yes | Unclear | Yes | Yes | Yes | Yes | Yes | NA |
| Choi Y 2013 | Yes | Yes | Unclear | Yes | Yes | Yes | Yes | Yes | NA |
| Asano N 2012 | Yes | Yes | Unclear | Yes | Yes | Yes | Yes | Yes | NA |
| Sumida T 2009 | Yes | No | Unclear | Yes | Yes | Yes | Yes | Yes | NA |
| Stathis A 2009 | Yes | No | Unclear | Yes | Yes | Yes | Yes | Yes | Yes |
| Chung S 2009 | Yes | Yes | Unclear | Yes | Yes | Yes | Yes | Yes | Yes |
| Yamamoto H 2008 | Yes | Yes | Unclear | Yes | Yes | Yes | Yes | Yes | NA |
| Todorovic M 2008 | Yes | Yes | Unclear | No | Yes | Yes | Yes | Yes | NA |
| Nakamura T 2008 | Yes | Yes | Unclear | Yes | Yes | Yes | Yes | Yes | Yes |
| Dong G 2008 | Yes | Yes | Unclear | Yes | Yes | Yes | Yes | Yes | NA |
| Nakamura S 2006 | Yes | Yes | Unclear | Yes | Yes | Yes | Yes | Yes | NA |
| Gisbert J 2006 | Yes | Yes | Unclear | Yes | Yes | Yes | Yes | Yes | NA |
| Cheng T 2006 | Yes | Yes | Unclear | Yes | Yes | Yes | Yes | Yes | NA |
| Akamatsu T 2006 | Yes | Yes | Unclear | Yes | Yes | Yes | Yes | Yes | NA |
| Lévy M 2005 | Yes | Yes | Unclear | Yes | Yes | Yes | Yes | Yes | NA |
| Chen L 2005 | Yes | Yes | Unclear | Yes | No | Yes | Yes | Yes | NA |
| Bao B 2005 | Yes | Yes | Unclear | Yes | Unclear | Yes | Yes | No | NA |
| Lee S 2004 | Yes | Yes | Unclear | Yes | Unclear | Yes | Yes | No | NA |
| Iwano M 2004 | Yes | Yes | Unclear | Yes | Yes | Yes | Yes | Yes | NA |
| Inagaki H 2004 | Yes | Yes | Unclear | Yes | Yes | Yes | Yes | Yes | NA |
| Yeh H 2003 | Yes | Yes | Unclear | Yes | No | Yes | Yes | No | NA |
| Lehours P 2003 | Yes | Yes | Unclear | Yes | No | Yes | Yes | No | NA |
| Goda K 2003 | Yes | Yes | Unclear | Yes | No | Yes | Yes | Yes | NA |
| Weston A 1999 | Yes | Yes | Unclear | Yes | No | Yes | Yes | No | NA |
| Steinbach G 1999 | Yes | Yes | Unclear | Yes | Yes | Yes | Yes | No | NA |

NA, not applicable; 1#, Was the sample frame appropriate to address the target population?; 2#, Were study participants sampled in an appropriate way?; 3#, Was the sample size adequate?; 4#, Were the study subjects and the setting described in detail?; 5#, Was the data analysis conducted with sufficient coverage of the identified sample?; 6#, Were valid methods used for the identification of the condition?; 7#, Was the condition measured in a standard, reliable way for all participants?; 8#, Was there appropriate statistical analysis?; 9#, Was the response rate adequate, and if not, was the low response rate managed appropriately?

**proportion of Helicobacter pylori-negative gastric mucosa-associated lymphoid tissue lymphoma**

**Figure S1. Proportion of H. pylori-negative gastric MALT lymphoma of all included studies**

**
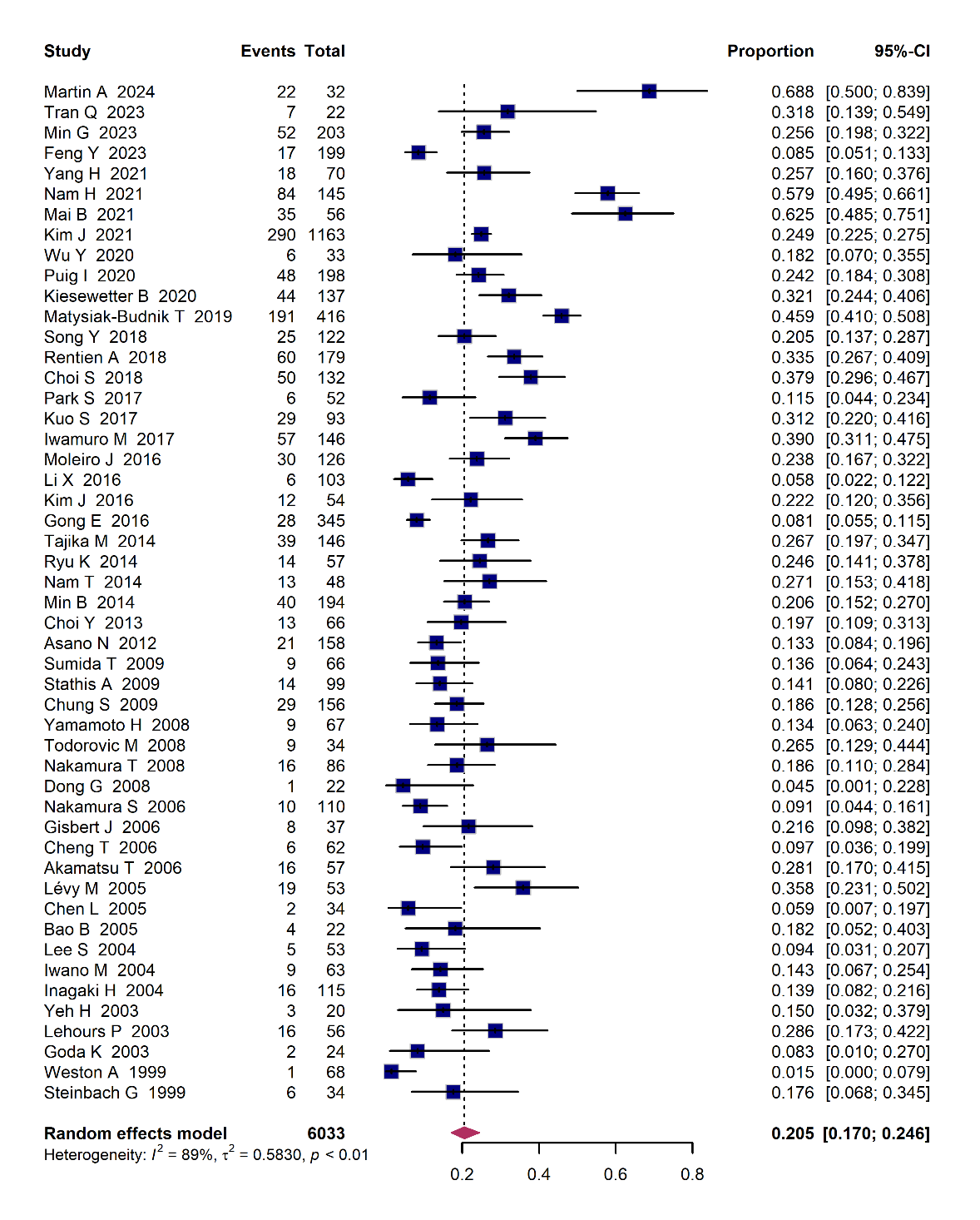
**

**Figure S2. Proportion of H. pylori-negative gastric MALT lymphoma in studies published in Asian countries**


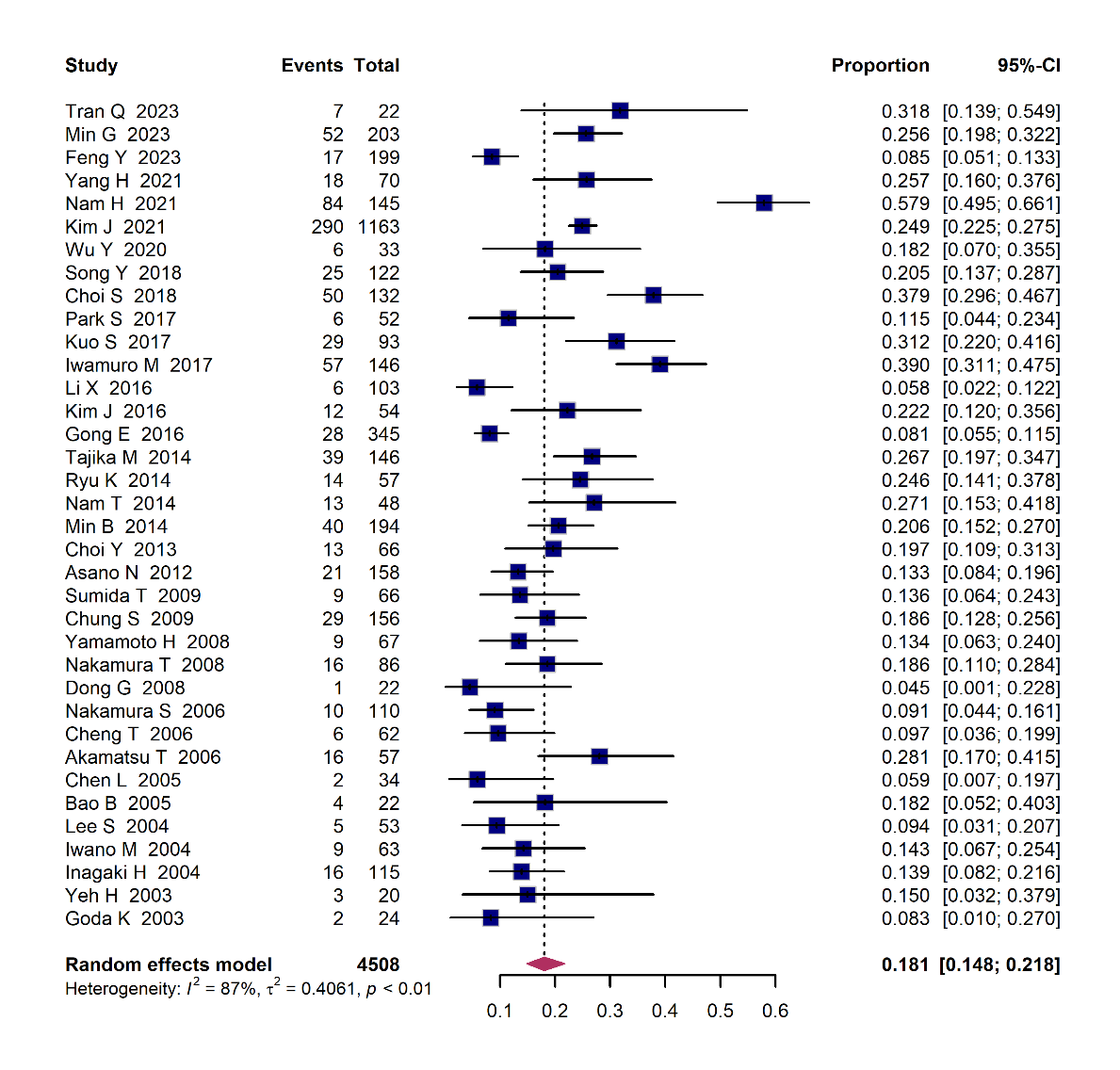


**Figure S3. Proportion of H. pylori-negative gastric MALT lymphoma in studies published in non-Asian countries**

**
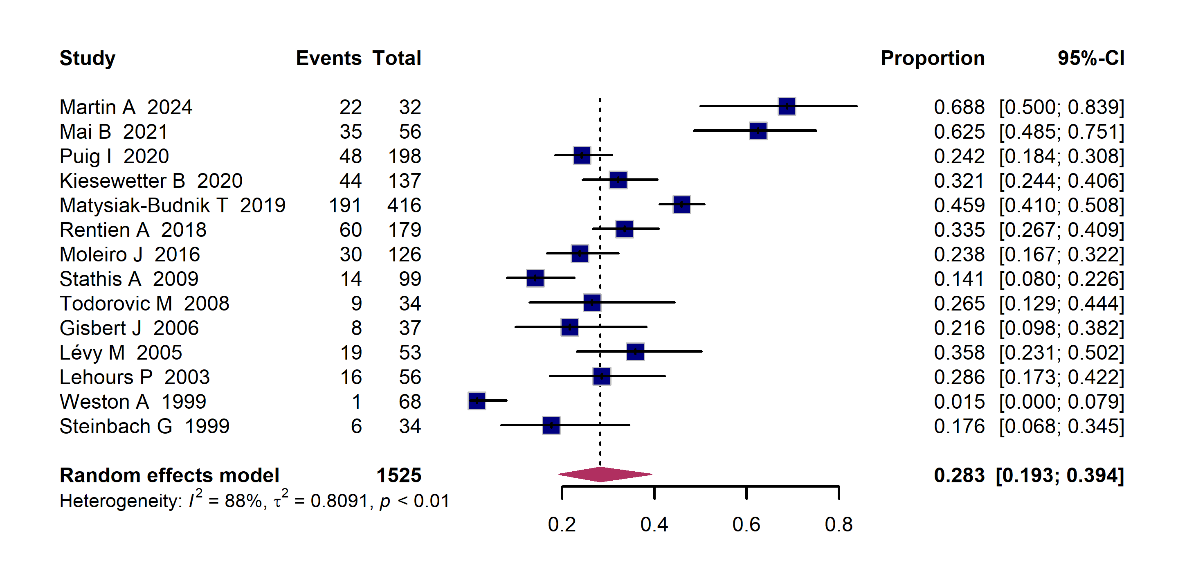
**

**Figure S4. Proportion of H. pylori-negative gastric MALT lymphoma in studies applying ≤ 3 H. pylori detection tests**


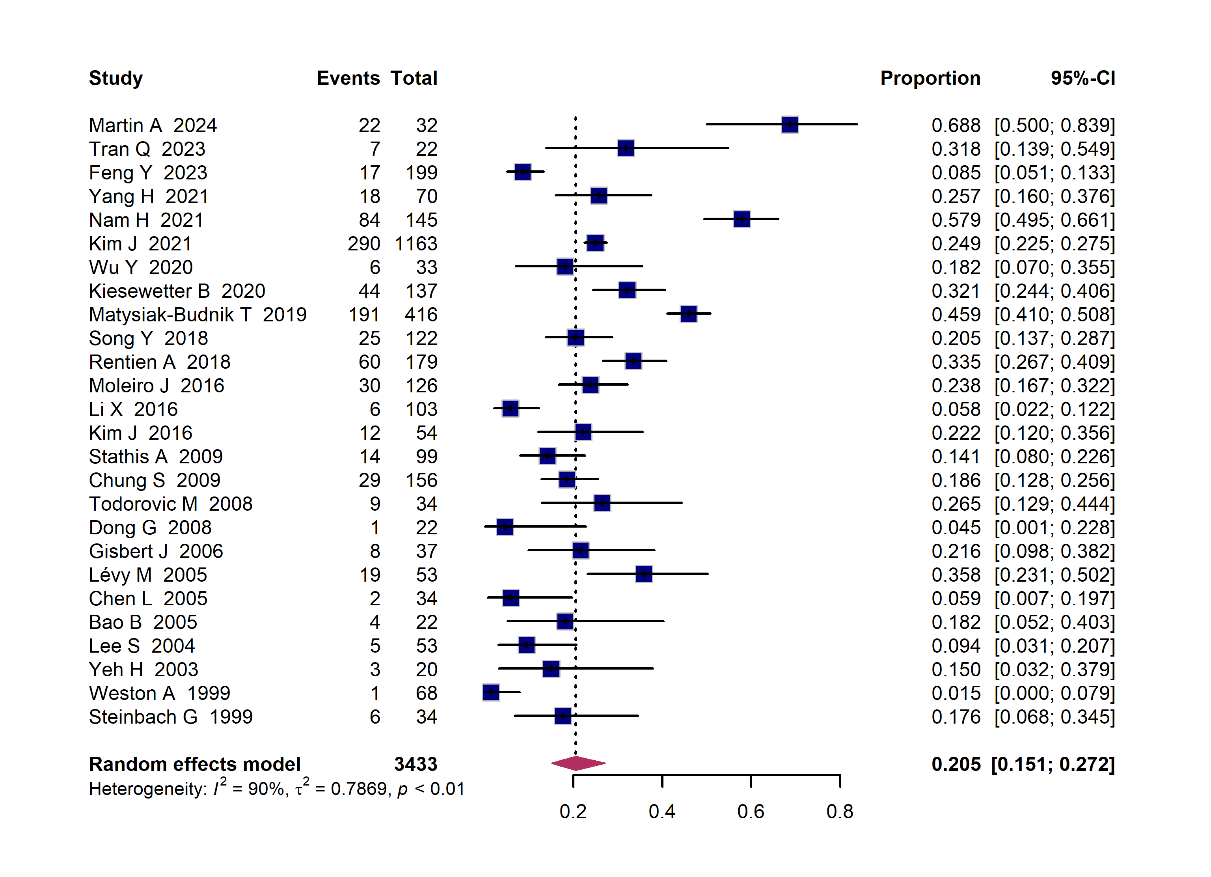


**Figure S5. Proportion of H. pylori-negative gastric MALT lymphoma in studies applying > 3 H. pylori detection tests**


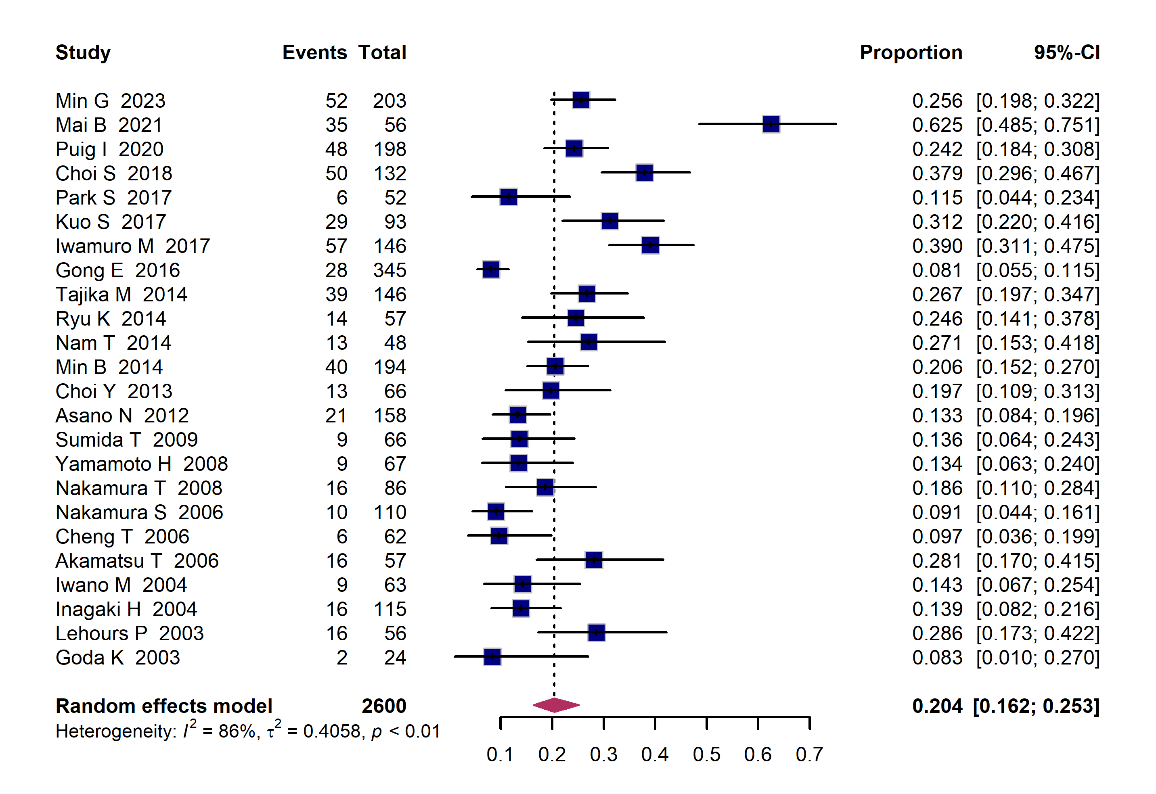


**Figure S6. Proportion of H. pylori-negative gastric MALT lymphoma in studies with a publication year ≤ 2013**


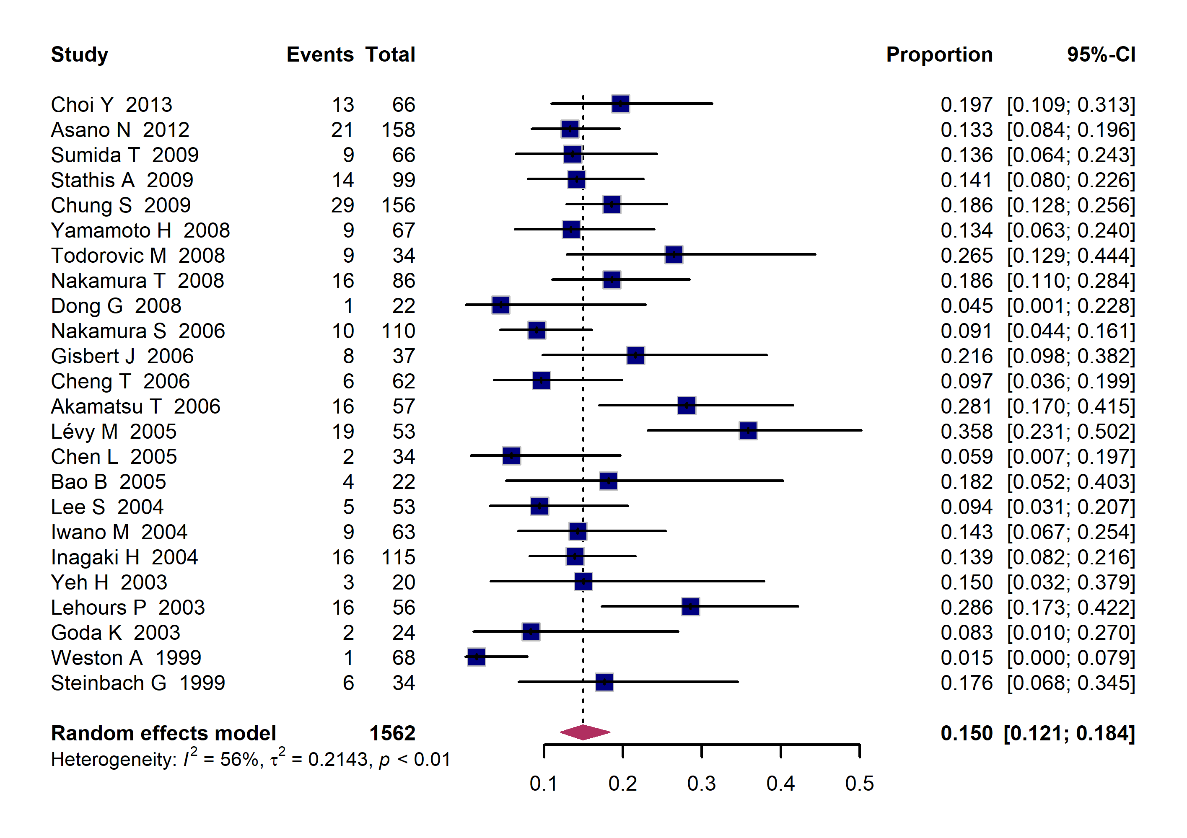


**Figure S7. Proportion of H. pylori-negative gastric MALT lymphoma in studies with a publication year > 2013**


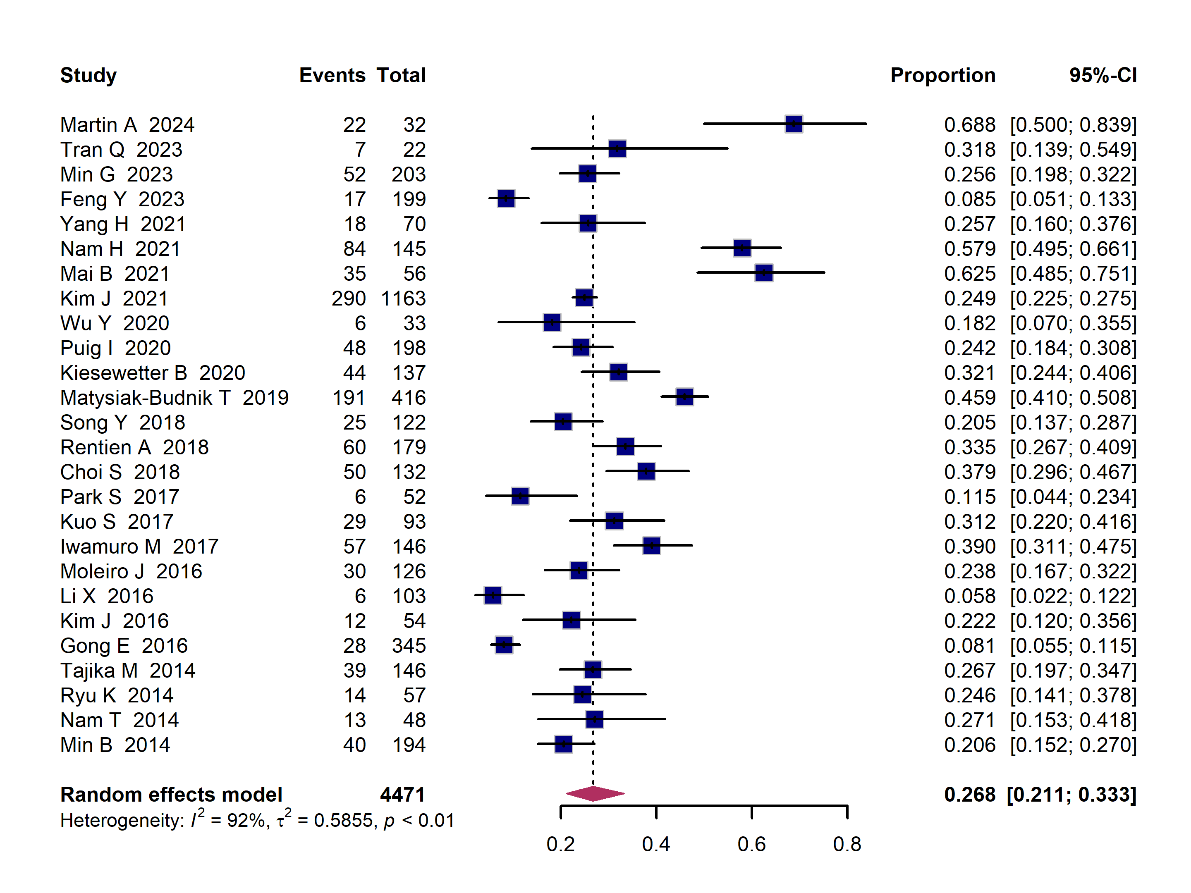


**Figure S8. Proportion of H. pylori-negative gastric MALT lymphoma in studies with a sample size ≤ 100 participants**

**
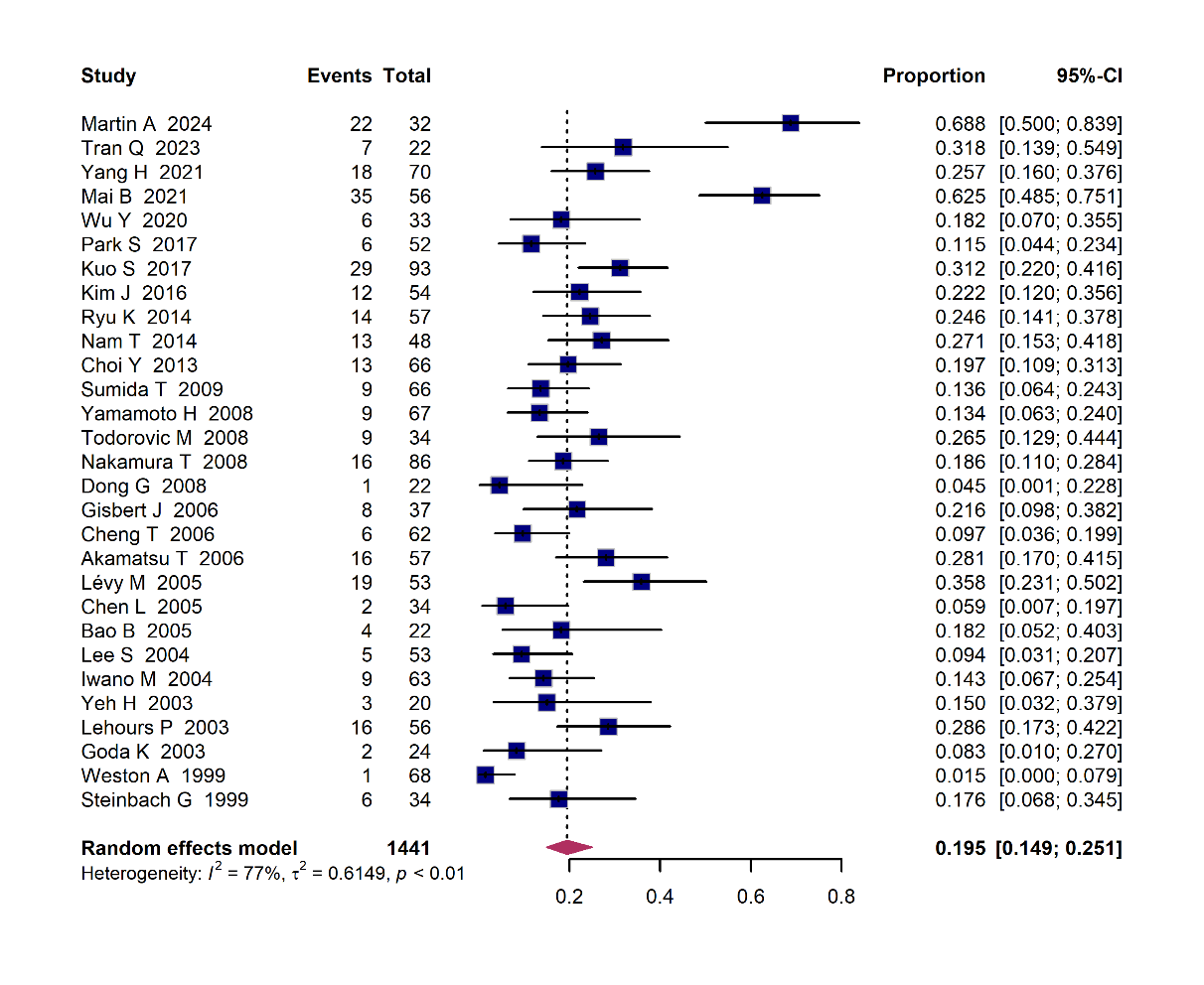
**

**Figure S9. Proportion of H. pylori-negative gastric MALT lymphoma in studies with a sample size >100 participants**

**
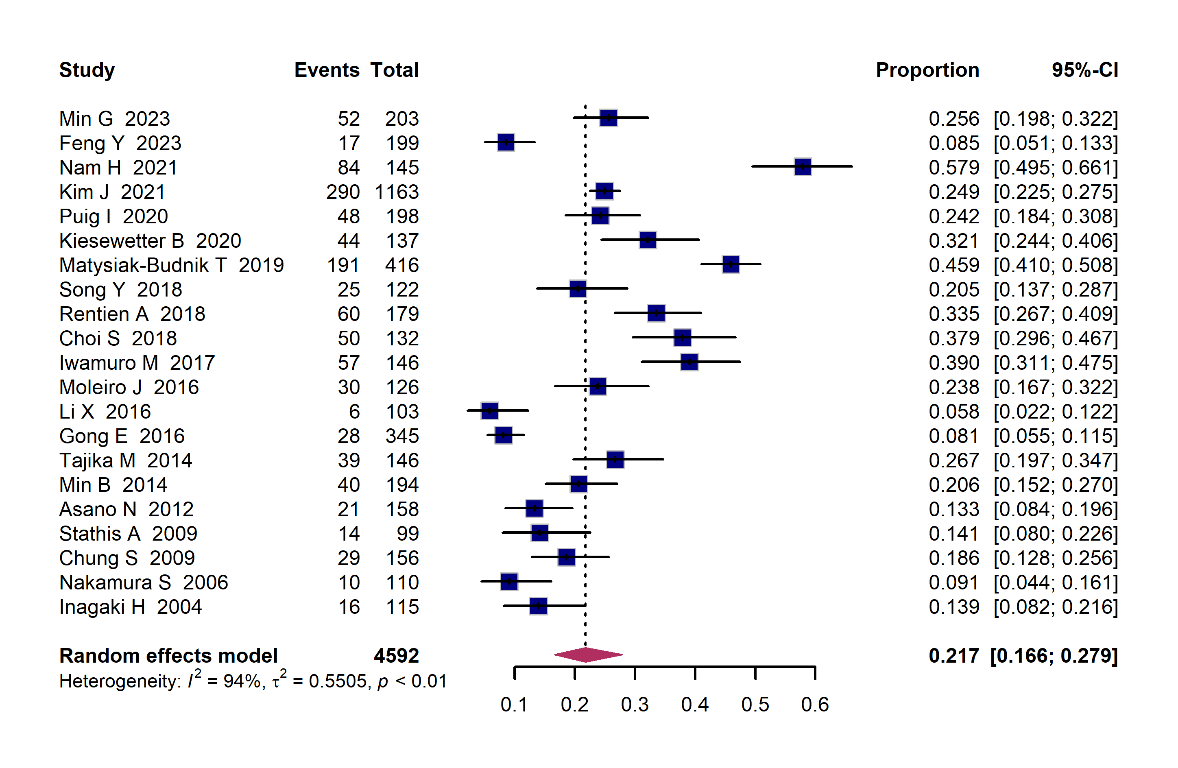
**

**Figure S10. Proportion of H. pylori-negative gastric MALT lymphoma in male patients**

**
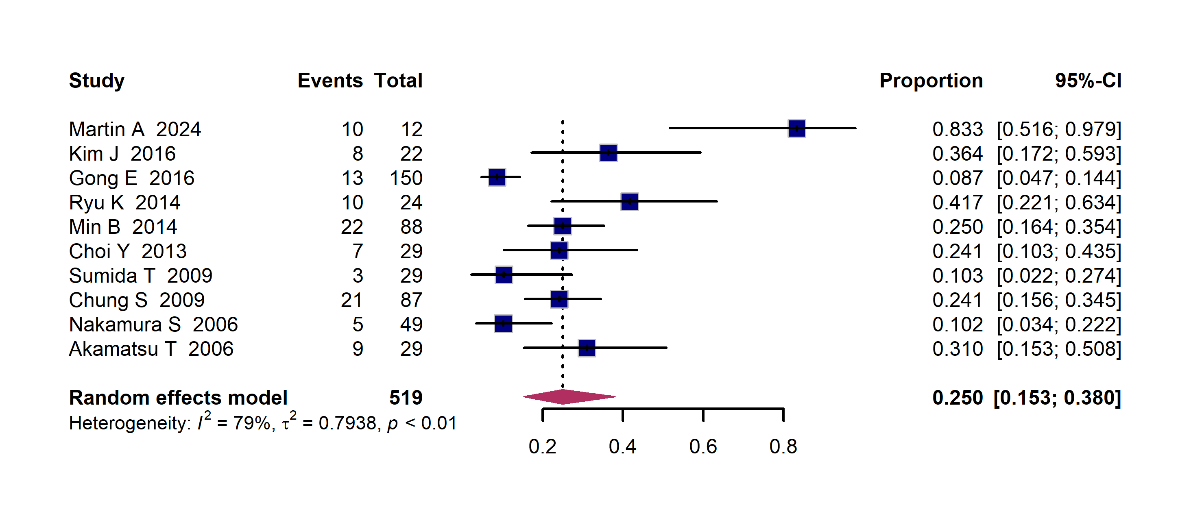
**

**Figure S11. Proportion of H. pylori-negative gastric MALT lymphoma in female patients**

**
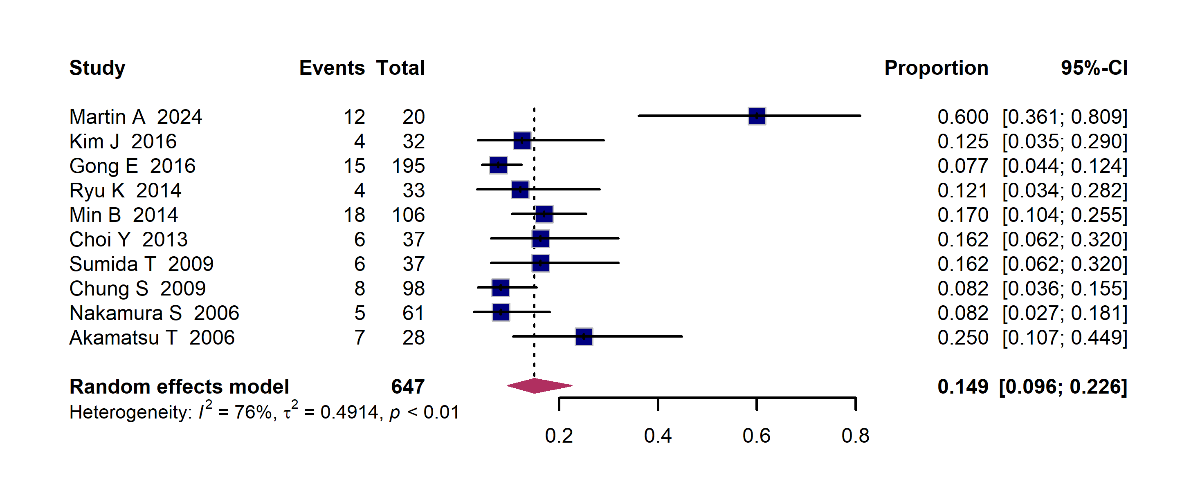
**

**Figure S12. Proportion of H. pylori-negative gastric MALT lymphoma in patients with distal lesions**

**
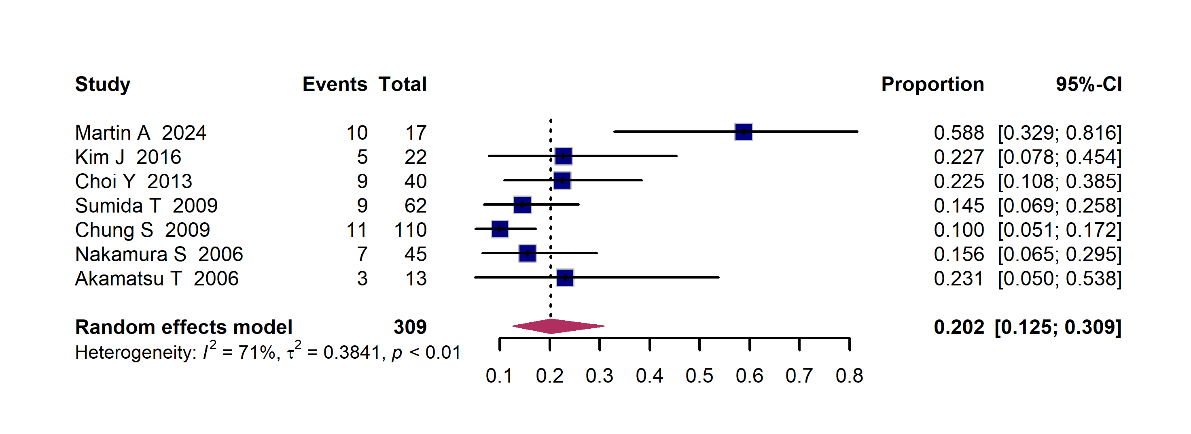
**

**Figure S13. Proportion of H. pylori-negative gastric MALT lymphoma in patients with proximal lesions**

**
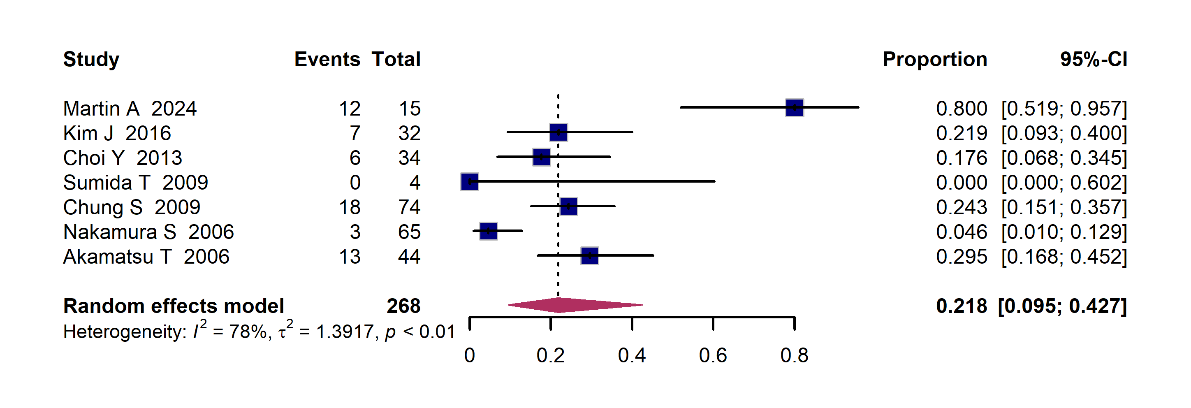
**

**Figure S14. Proportion of H. pylori-negative gastric MALT lymphoma in patients with single lesion**

**
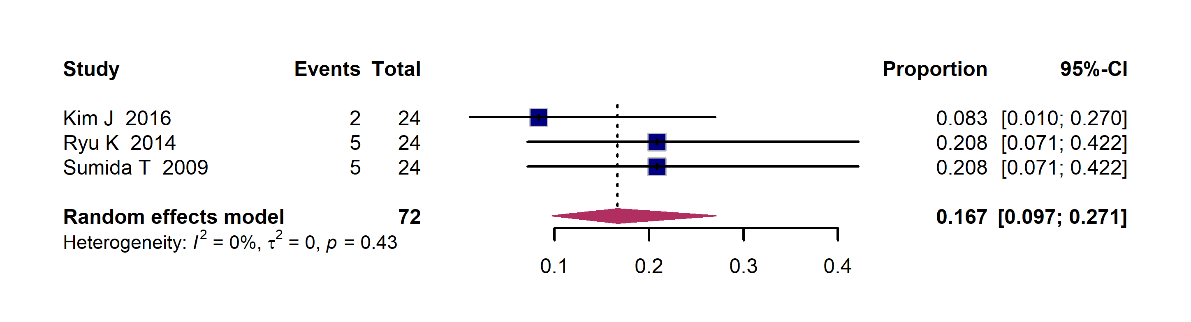
**

**Figure S15. Proportion of H. pylori-negative gastric MALT lymphoma in patients with multiple lesions**

**
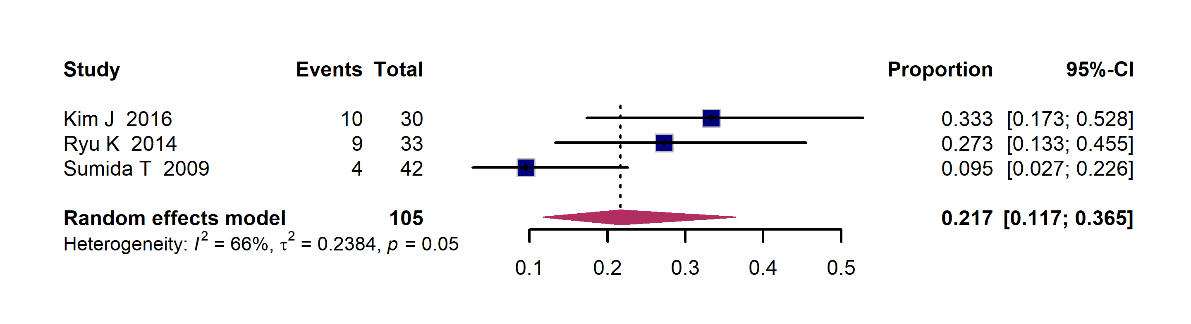
**

**Figure S16. Proportion of H. pylori-negative gastric MALT lymphoma in patients with endoscopic morphology of superficial type**

**
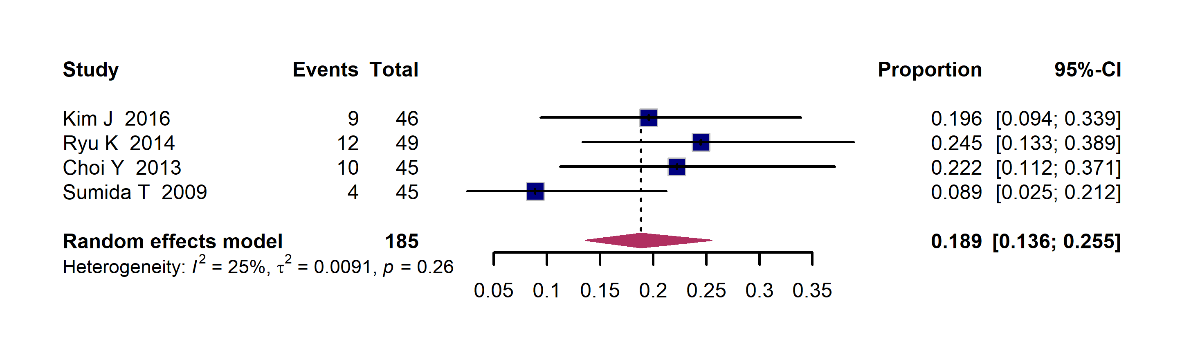
**

**Figure S17. Proportion of H. pylori-negative gastric MALT lymphoma in patients with endoscopic morphology of non-superficial type**

**
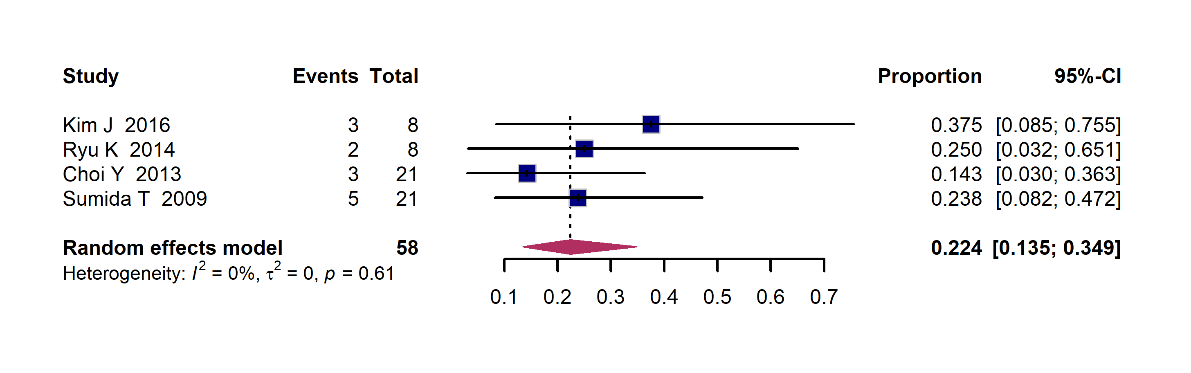
**

**Figure S18. Proportion of H. pylori-negative gastric MALT lymphoma in patients with mucosal invasion**

**
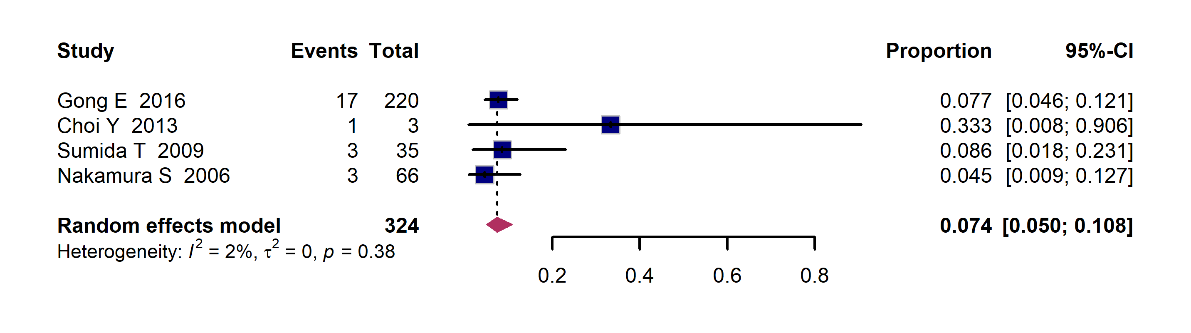
**

**Figure S19. Proportion of H. pylori-negative gastric MALT lymphoma in patients with submucosal (or beyond) invasion**

**
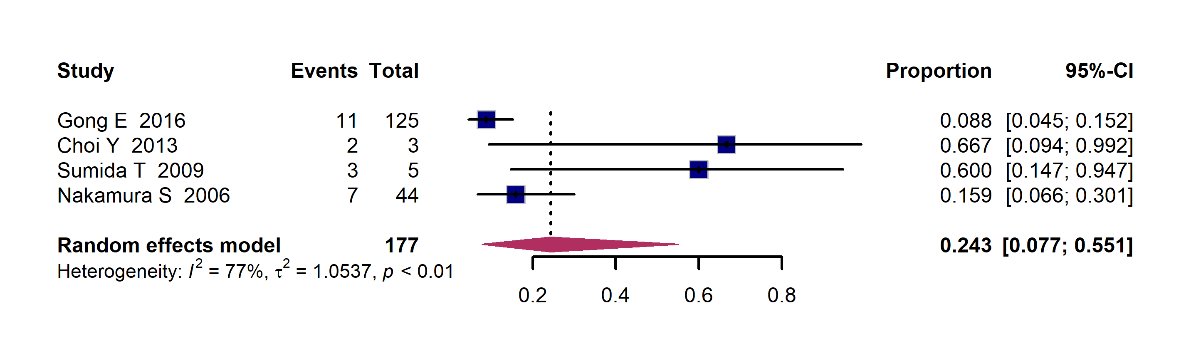
**

**Figure S20. Proportion of H. pylori-negative gastric MALT lymphoma in patients with Lugano stage I**

**
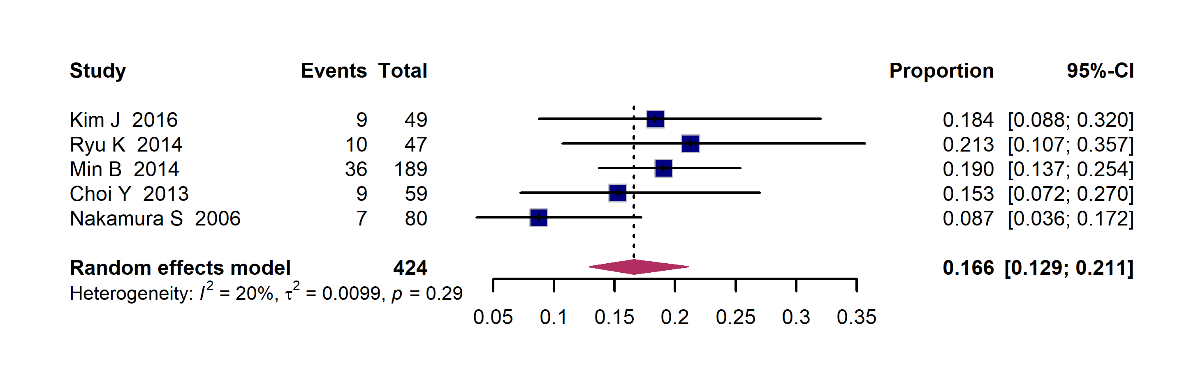
**

**Figure S21. Proportion of H. pylori-negative gastric MALT lymphoma in patients with Lugano stage II or more**

**
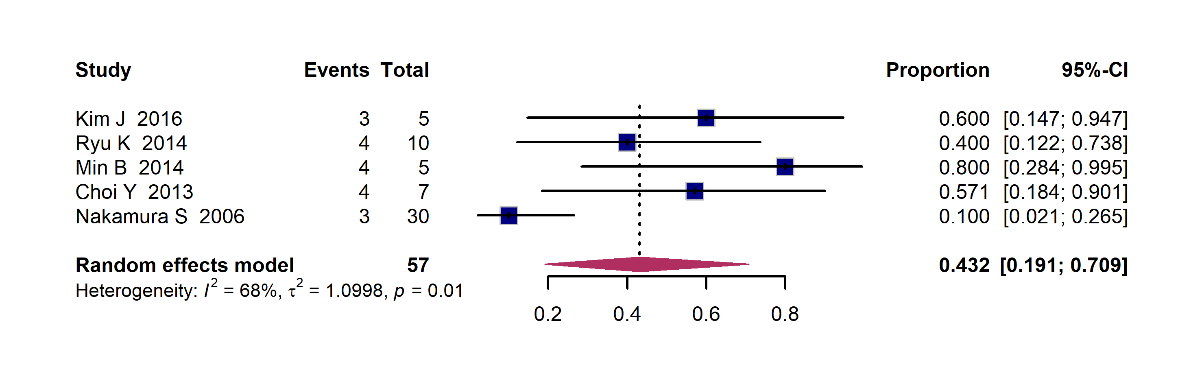
**

**Figure S22. Proportion of H. pylori-negative gastric MALT lymphoma in patients with modified Ann Arbor stage IE**

**
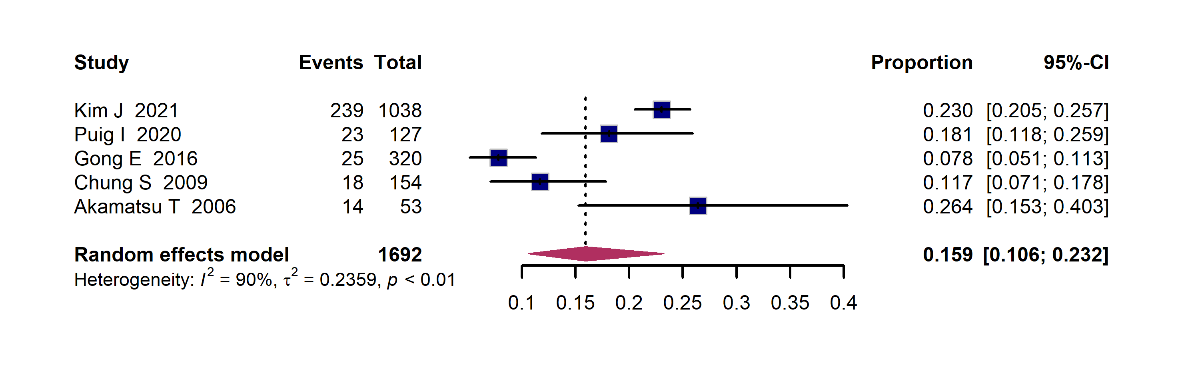
**

**Figure S23. Proportion of H. pylori-negative gastric MALT lymphoma in patients with modified Ann Arbor stage IIE**

**
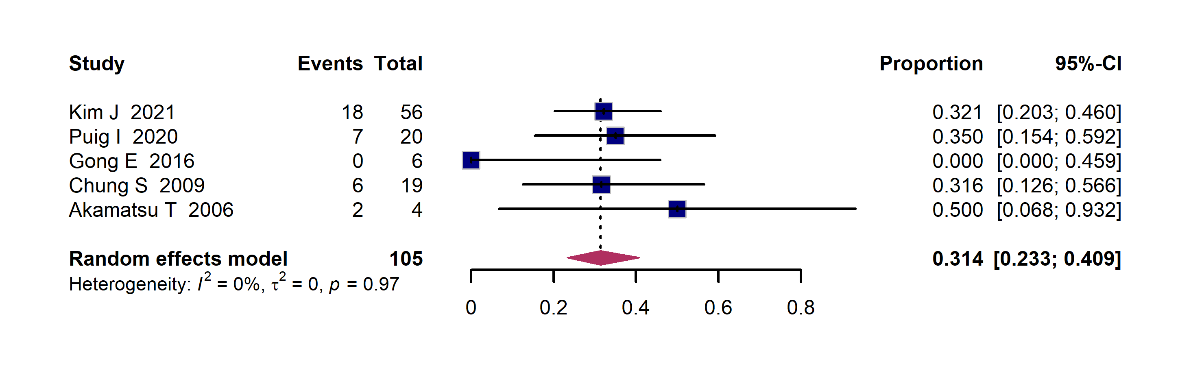
**

**Figure S24. Proportion of H. pylori-negative gastric MALT lymphoma in patients with modified Ann Arbor stage IIIE/IV**

**
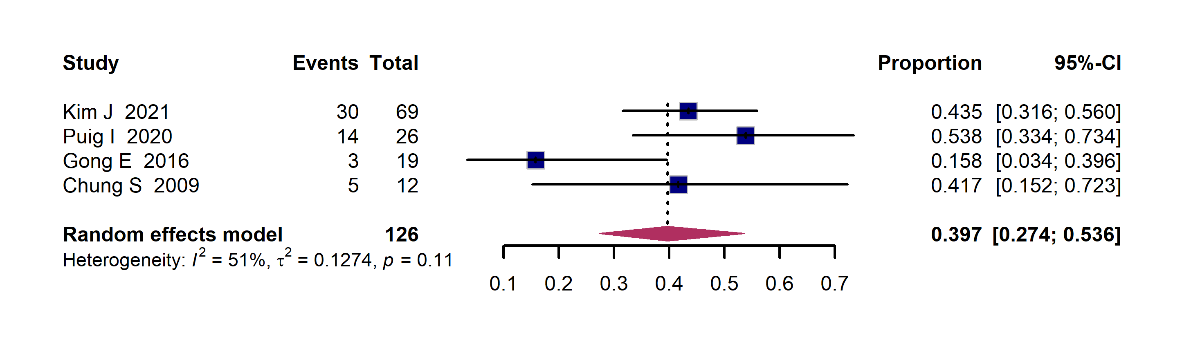
**

**Table S3.** **Pooled proportion of H. pylori-negative gastric MALT lymphoma based on different countries/regions and relevant World Bank income level**

| **Population** | **Studies**  **(n)** | **Patients**  **(n)** | **Proportion**  **(95% CI)** | **I^2^**  **(%)** | **P**  **value** |
| --- | --- | --- | --- | --- | --- |
| ***Countries/regions*** | | | | | |
| France | 5 | 736 | 0.412  (0.309-0.524) | 81 | <0.01 |
| Vietnam | 1 | 22 | 0.318  (0.139-0.549) | - | - |
| Korea | 13 | 2668 | 0.221  (0.161-0.294) | 92 | <0.01 |
| China | 7 | 571 | 0.132  (0.084-0.202) | 74 | <0.01 |
| America | 3 | 158 | 0.154  (0.019-0.636) | 94 | <0.01 |
| Spain | 2 | 235 | 0.238  (0.188-0.297) | 0 | 0.73 |
| Austria | 1 | 137 | 0.321  (0.244-0.406) | - | - |
| Taiwan | 4 | 209 | 0.142  (0.067-0.275) | 79 | <0.01 |
| Japan | 11 | 1038 | 0.173  (0.128-0.230) | 83 | <0.01 |
| Portugal | 1 | 126 | 0.238  (0.167-0.322) | - | - |
| Switzerland | 1 | 99 | 0.141  (0.080-0.226) | - | - |
| Serbia | 1 | 34 | 0.265  (0.128-0.444) | - | - |
| ***World Bank income level**** | | | | | |
| High-income  economies | 41 | 5406 | 0.216  (0.176-0.263) | 90 | <0.01 |
| Middle-income  economies | 9 | 627 | 0.157  (0.105-0.228) | 73 | <0.01 |

H. pylori, Helicobacter pylori; MALT, mucosa-associated lymphoid tissue; CI, confidence interval.

*, the following countries/regions belong to high-income economies: France, Korea, America, Spain, Austria, Taiwan, Japan, Portugal, and Switzerland; the following countries belong to middle-income economies: Vietnam, China, and Serbia.

**Table S4. Pooled proportion of H. pylori-negative gastric MALT lymphoma based on different diagnostic tests for H. pylori**

| **Population** | **Studies**  **(n)** | **Patients**  **(n)** | **Proportion**  **(95% CI)** | **I^2^**  **(%)** | **P**  **value** |
| --- | --- | --- | --- | --- | --- |
| ***Two diagnostic tests for H. pylori*** | | | | | |
| Histology, PCR | 2 | 211 | 0.495  (0.258-0.734) | 92 | <0.01 |
| Histology, culture | 2 | 87 | 0.322  (0.232-0.427) | 0 | 0.36 |
| Histology, rapid urease test | 2 | 209 | 0.163  (0.119-0.219) | 57 | 0.13 |
| Histology, urea breath test | 4 | 243 | 0.158  (0.087-0.270) | 75 | <0.01 |
| Histology, serology | 2 | 205 | 0.083  (0.007-0.546) | 91 | <0.01 |
| Rapid urease test, urea breath test | 2 | 221 | 0.155  (0.056-0.360) | 89 | <0.01 |
| ***Three diagnostic tests for H. pylori*** | | | | | |
| Histology, PCR, rapid urease test | 1 | 22 | 0.182  (0.052-0.403) | - | - |
| Histology, culture, rapid urease test | 1 | 34 | 0.059  (0.007-0.197) | - | - |
| Histology, culture, urea breath test | 2 | 146 | 0.226  (0.165-0.301) | 0 | 0.39 |
| Histology, rapid urease test, urea breath test | 3 | 1339 | 0.244  (0.222-0.268) | 0 | 0.52 |
| Histology, rapid urease test, serology | 1 | 34 | 0.176  (0.068-0.345) | - | - |
| Histology, urea breath test, serology | 4 | 682 | 0.263  (0.093-0.554) | 94 | <0.01 |
| ***Four diagnostic tests for H. pylori*** | | | | | |
| Histology, PCR, culture, serology | 1 | 56 | 0.286  (0.173-0.422) | - | - |
| Histology, culture, rapid urease test, serology | 1 | 62 | 0.097  (0.036-0.199) | - | - |
| Histology, culture, urea breath test, serology | 1 | 57 | 0.281  (0.170-0.415) | - | - |
| Histology, rapid urease test, urea breath test, serology | 10 | 1211 | 0.192  (0.140-0.258) | 87 | <0.01 |
| Histology, urea breath test, stool antigen, serology | 1 | 56 | 0.625  (0.485-0.751) | - | - |
| ***Five diagnostic tests for H. pylori*** | | | | | |
| Histology, PCR, rapid urease test, urea breath test, serology | 1 | 203 | 0.256  (0.198-0.322) | - | - |
| Histology, culture, rapid urease test, urea breath test, serology | 7 | 611 | 0.152  (0.111-0.204) | 65 | <0.01 |
| Histology, rapid urease test, urea breath test, stool antigen, serology | 1 | 146 | 0.390  (0.311-0.475) | - | - |
| ***Six diagnostic tests for H. pylori*** | | | | | |
| Histology, culture, rapid urease test, urea breath test, stool antigen, serology | 1 | 198 | 0.242  (0.184-0.308) | - | - |

H. pylori, Helicobacter pylori; MALT, mucosa-associated lymphoid tissue; CI, confidence interval.

**CHARACTERISTICS OF HELICOBACTER PYLORI-NEGATIVE GASTRIC MUCOSA-ASSOCIATED LYMPHOID TISSUE LYMPHOMA**

**Figure S25. Association of patients’age with H. pylori-negative gastric MALT lymphoma**

**
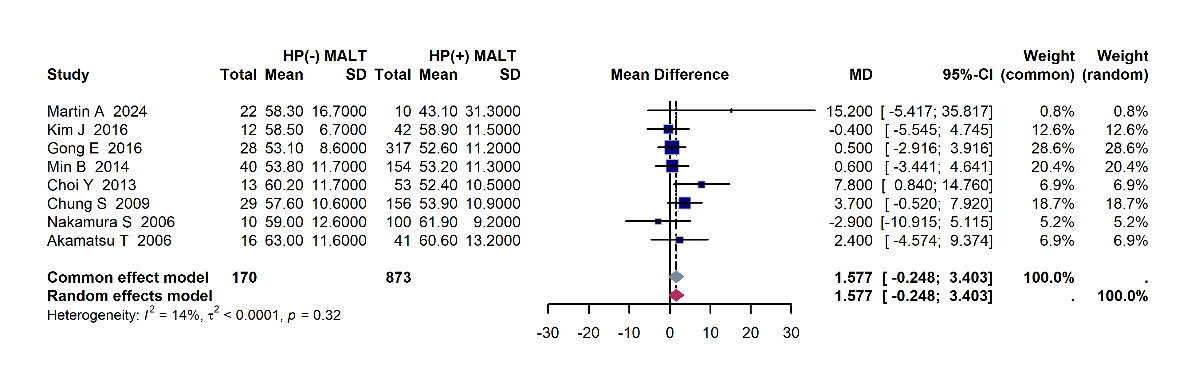
**

**Figure S26. Association of male gender with H. pylori-negative gastric MALT lymphoma**

**
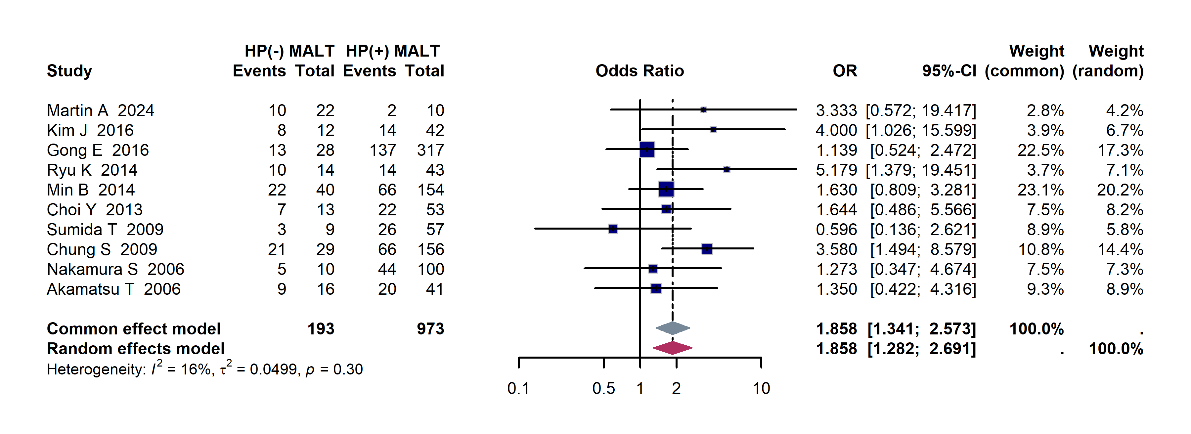
**

**Figure S27. Association of proximal lesions with H. pylori-negative gastric MALT lymphoma**

**
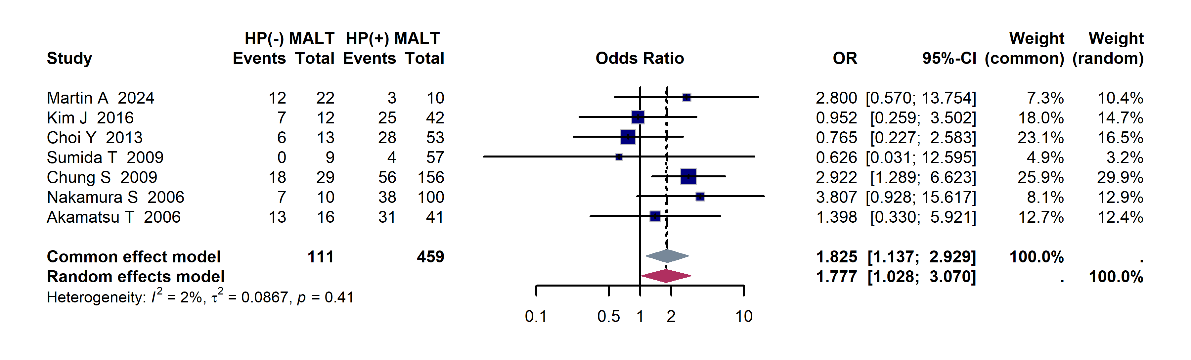
**

**Figure S28. Association of multiple lesions with H. pylori-negative gastric MALT lymphoma**

**
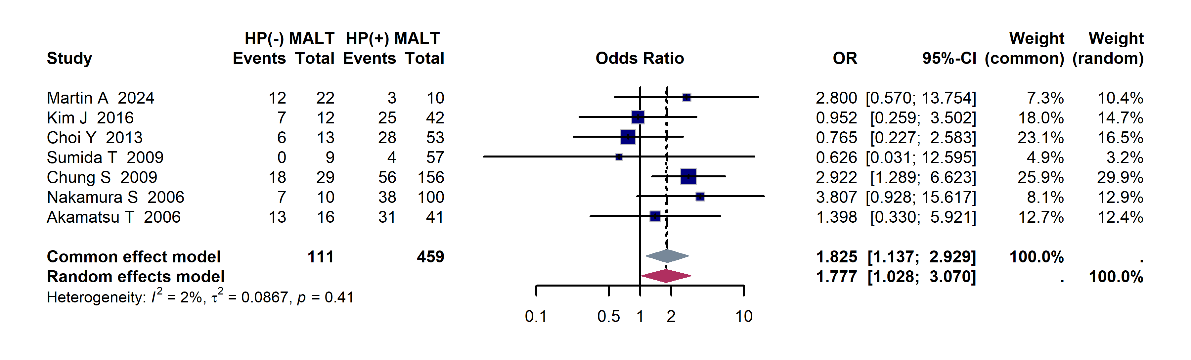
**

**Figure S29. Association of superficial type morphology with H. pylori-negative gastric MALT lymphoma**

**
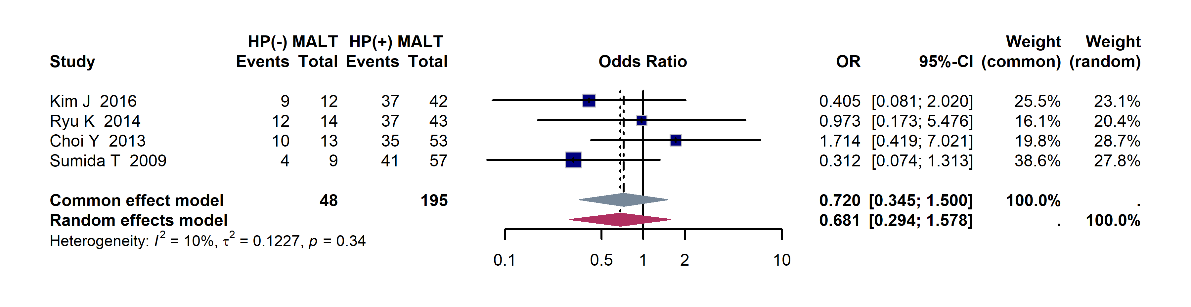
**

**Figure S30. Association of submucosal (or beyond) invastion with H. pylori-negative gastric MALT lymphoma**

**
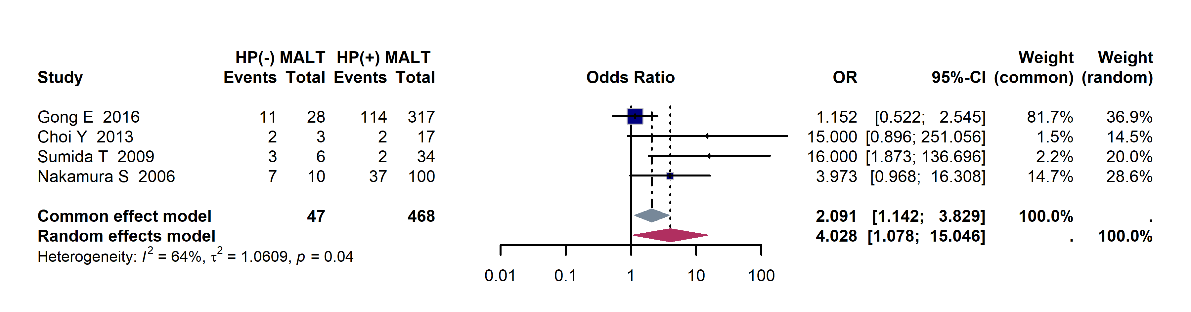
**

**Figure S31. Association of t(11;18)(q21;q21) positivity with H. pylori-negative gastric MALT lymphoma**

**
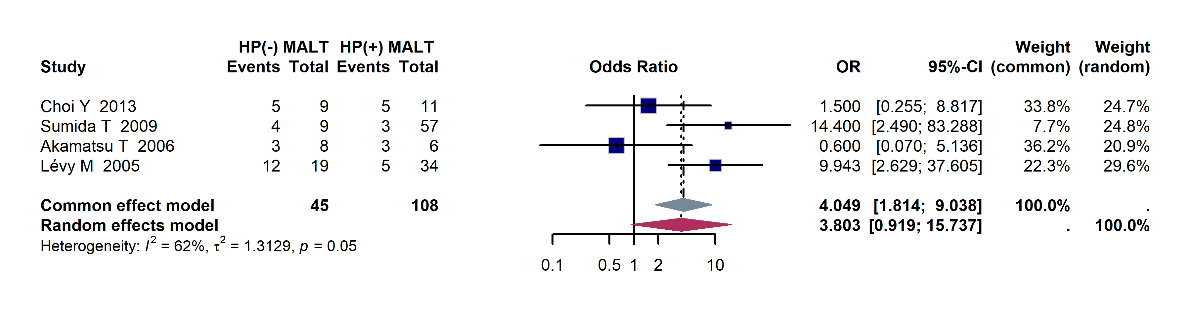
**

**Figure S32. Association of clinical stage (Lugano stage I) with H. pylori-negative gastric MALT lymphoma**

**
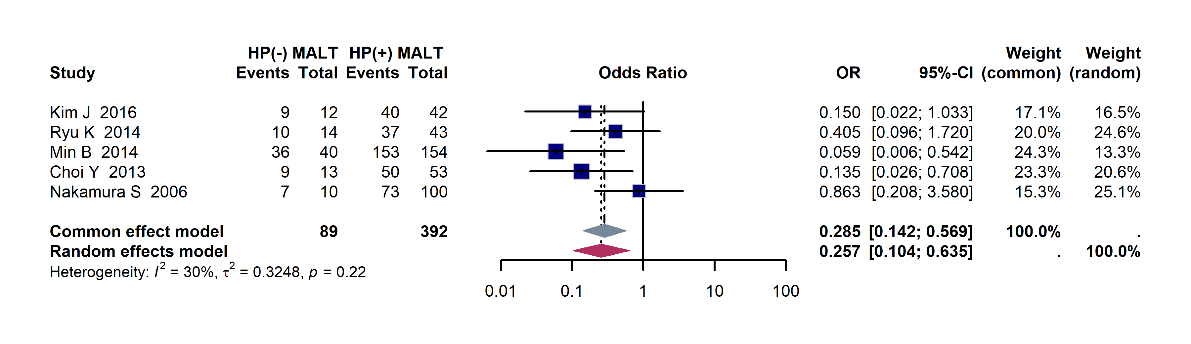
**

**Figure S33. Association of clinical stage (Lugano stage II or more) with H. pylori-negative gastric MALT lymphoma**

**
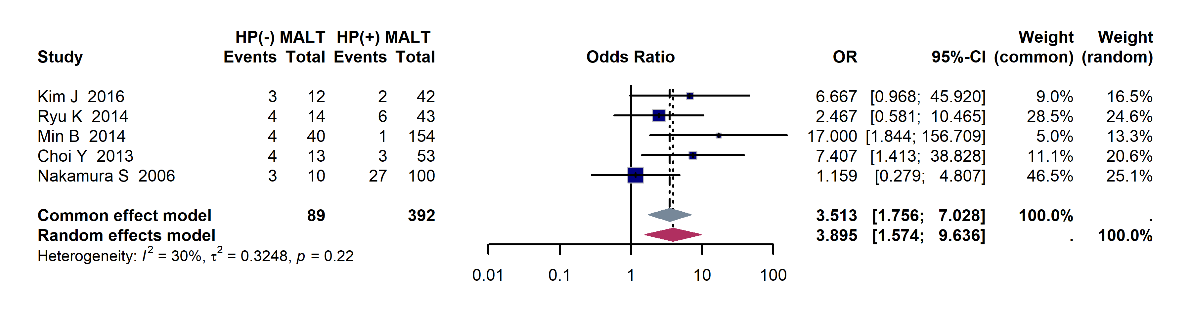
**

**Figure S34. Association of clinical stage (modified Ann Arbor stage IE) with H. pylori-negative gastric MALT lymphoma**

**
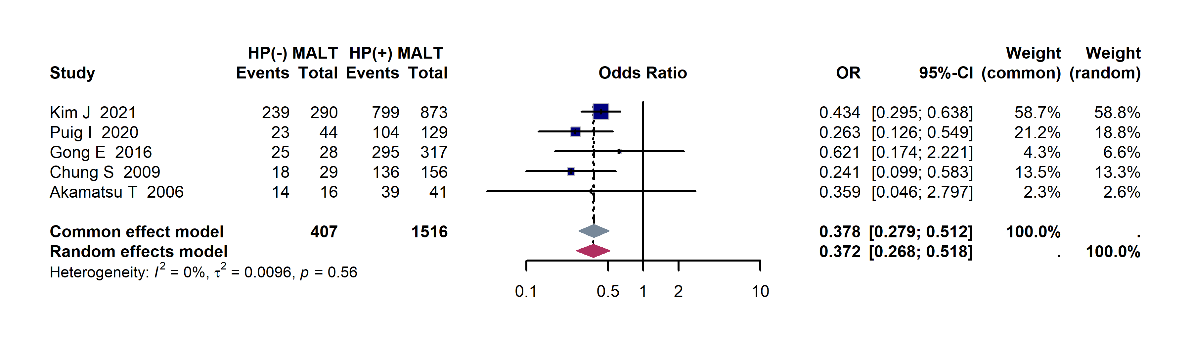
**

**Figure S35. Association of clinical stage (modified Ann Arbor stage IIE) with H. pylori-negative gastric MALT lymphoma**

**
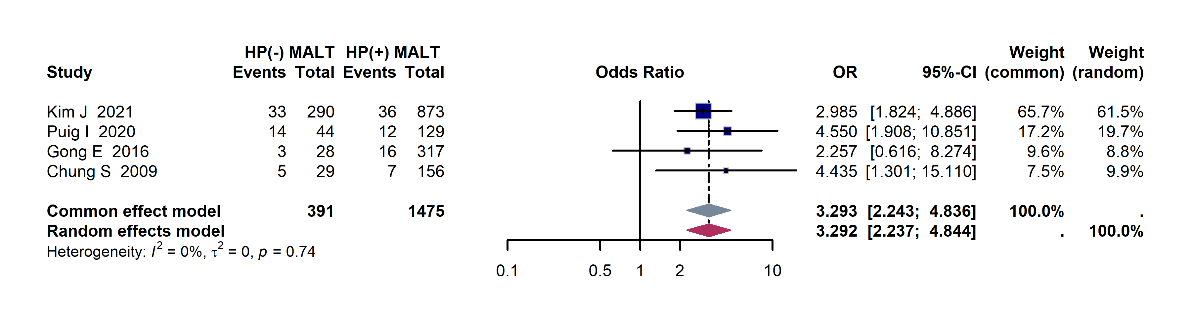
**

**Figure S36. Association of clinical stage (modified Ann Arbor stage IIIE/IV) with H. pylori-negative gastric MALT lymphoma**

**
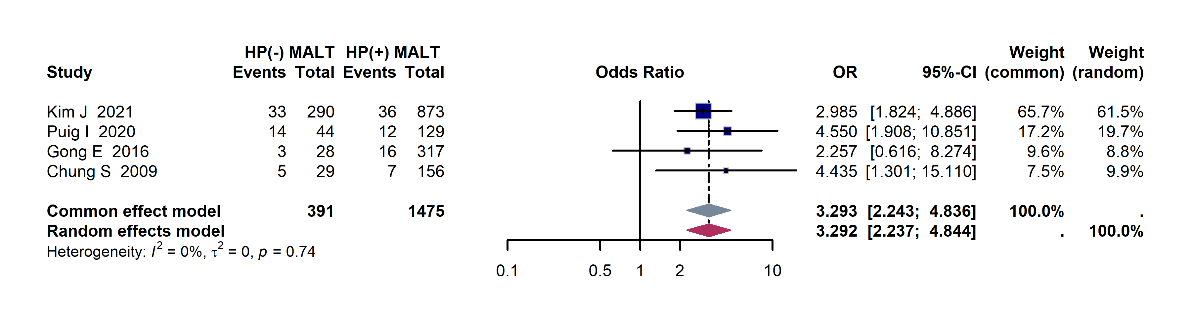
**

**HETEROGENEITY, TEMPORAL TREND, AND PUBLICATION BIAS**

**Table S5. Meta-regression analysis** **on the proportion of H. pylori-negative gastric MALT lymphoma**

|  |  | **Univariate**  **meta-regression** | | **Multivariate**  **meta-regression*** | |
| --- | --- | --- | --- | --- | --- |
| **Moderators** | **Studies, n** | **Coefficient**  **(95% CI)** | **P value** | **Coefficient**  **(95% CI)** | **P value** |
| ***Country of origin*** |  |  |  |  |  |
| Asian | 36 | Reference |  | Reference |  |
| Non-Asian | 14 | 0.643  (0.157 to 1.130) | 0.011 | 0.654  (0.225 to 1.083) | 0.004 |
| ***H. pylori detection*** |  |  |  |  |  |
| ≤ 3 tests | 26 | Reference |  |  |  |
| > 3 tests | 24 | -0.083  (-0.557 to 0.391) | 0.726 | - | - |
| ***Publication year*** |  |  |  |  |  |
| ≤ 2013 | 24 | Reference |  | Reference |  |
| >2013 | 26 | 0.707  (0.279 to 1.136) | 0.002 | 0.707  (0.313 to 1.100) | 0.001 |
| ***Sample size*** |  |  |  |  |  |
| ≤ 100 participants | 29 | Reference |  |  |  |
| >100 participants | 21 | 0.072  (-0.403 to 0.547) | 0.762 | - | - |

CI, confidence interval.

*, Multivariate meta-regression was performed when the univariate meta-regression P value was＜0.1. Finally, the R^2^ equals to 0.36.

**Figure S37. Temporal trend for the proportion of H. pylori-negative gastric MALT lymphoma based on publication year**

**
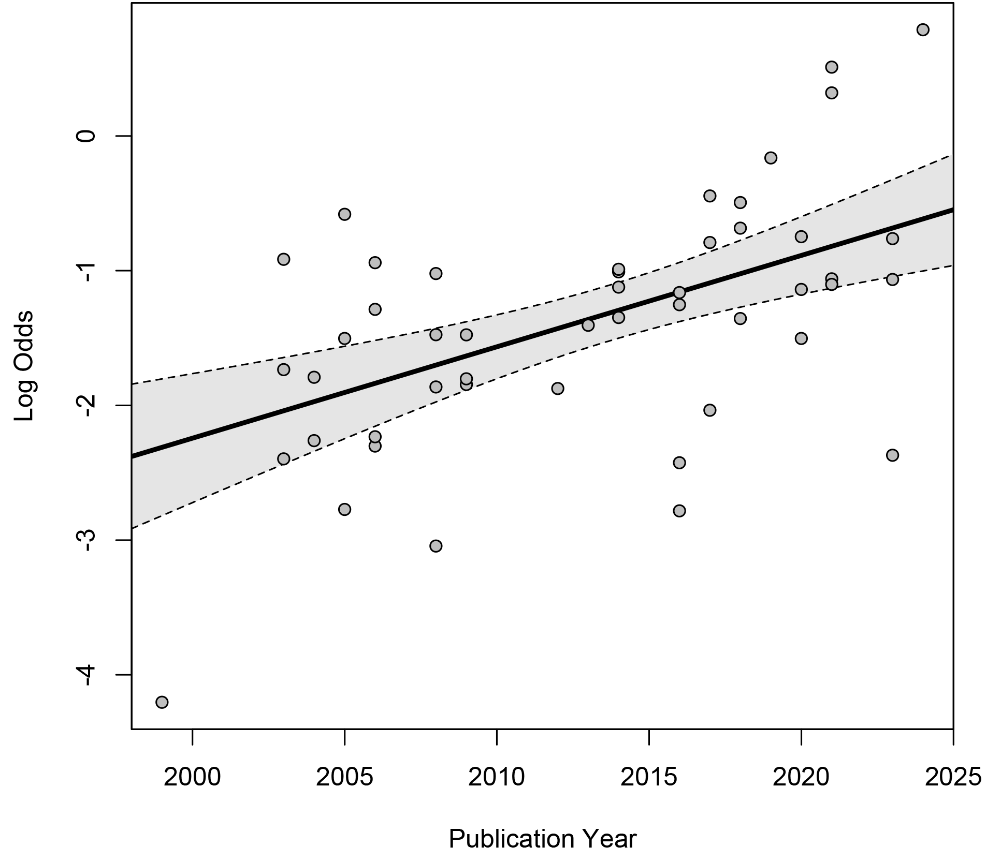
**

**Figure S38. Temporal trend for the proportion of H. pylori-negative gastric MALT lymphoma based on inclusion year**

**
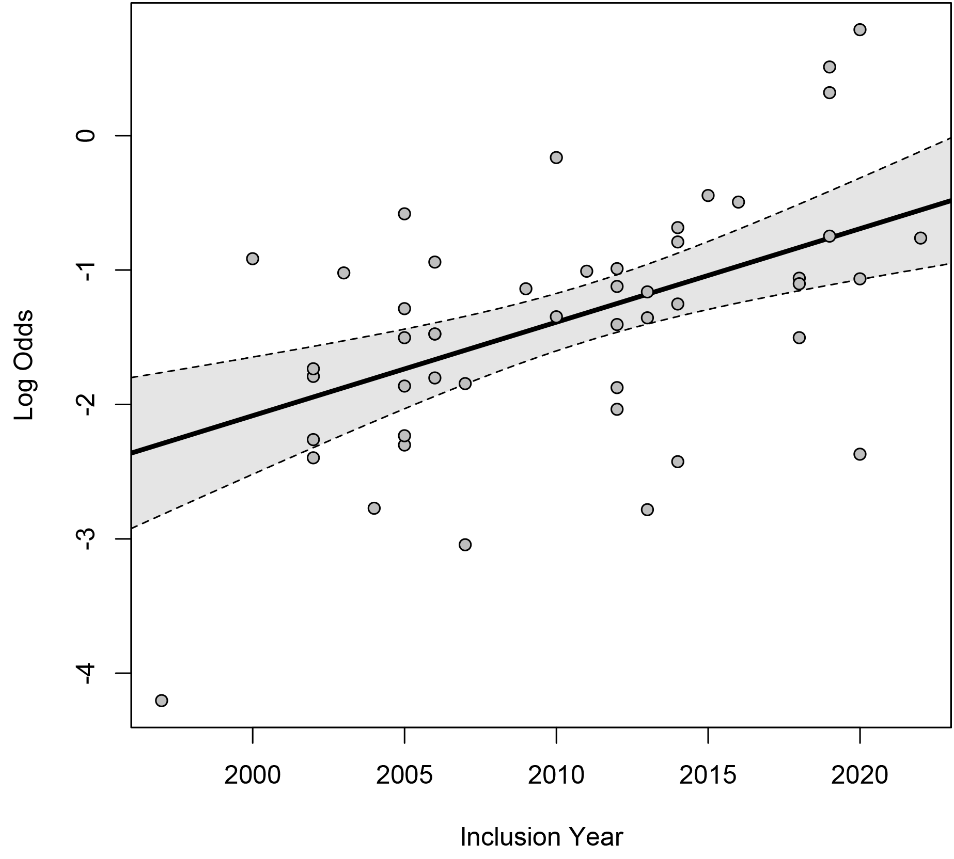
**

**Figure S39. Funnel plot for the proportion of H. pylori-negative gastric MALT lymphoma**

**
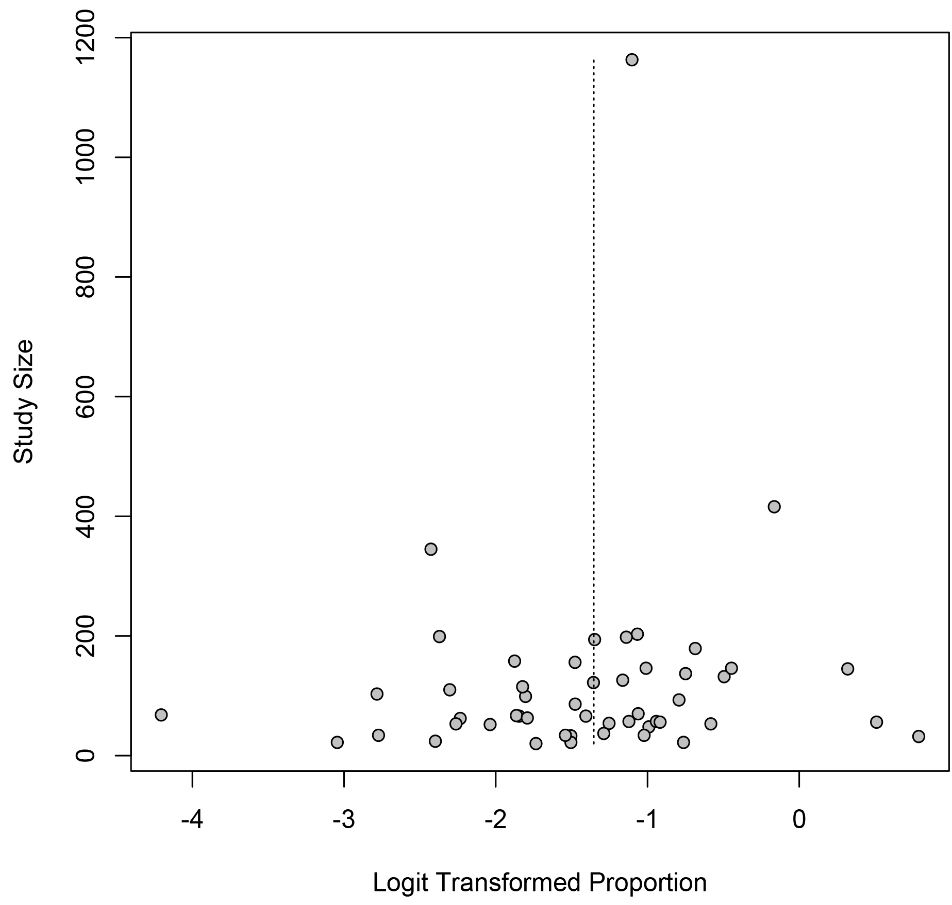
**
